# CLPTM1L interacts with ERLIN2 to stabilize SREBP1 and drive tumorigenesis in nasopharyngeal carcinoma

**DOI:** 10.1038/s41419-025-07635-8

**Published:** 2025-06-23

**Authors:** Jian Zhang, Shu-Qiang Liu, Xiang-Yu Xiong, Ya-Qing Zhou, Pan-Pan Wei, Xin-Yuan Guan, Jin-Xin Bei, Qian Cui, Chun-Ling Luo

**Affiliations:** 1 The Eighth Affiliated Hospital, Sun Yat-sen University, Shenzhen, China; 2 State Key Laboratory of Oncology in South China, Guangdong Key Laboratory of Nasopharyngeal Carcinoma Diagnosis and Therapy, Guangdong Provincial Clinical Research Center for Cancer, Collaborative Innovation Center for Cancer Medicine, Sun Yat-sen University Cancer Center, Guangzhou, China; 3 Department of Clinical Oncology, The University of Hong Kong, Hong Kong, China; 4 Department of Medical Oncology, National Cancer Centre Singapore, Singapore, Singapore; 5 Department of Pathology, Guangdong Provincial People’s Hospital (Guangdong Academy of Medical Sciences), Southern Medical University, Guangzhou, Guangdong China

**Keywords:** Oncogenes, Head and neck cancer

## Abstract

CLPTM1L has been identified as a susceptibility gene associated with nasopharyngeal carcinoma (NPC) risk, but its biological function and underlying mechanisms remain unclear. Here, we demonstrate that CLPTM1L is highly expressed in NPC patients and is associated with poor prognosis. Functional assays reveal that CLPTM1L overexpression significantly enhances NPC cell proliferation, migration and invasion, whereas its knockdown induces apoptosis and suppresses tumor growth. We further uncover that the transcription factor KLF1 directly binds to the promoter of CLPTM1L, driving its transcriptional activation. Transcriptome analysis indicates that CLPTM1L regulates various lipid metabolic pathways, notably upregulating SREBP1, a key metabolic regulator, thereby increasing intracellular free fatty acid levels in NPC cells. Mechanically, CLPTM1L interacts with the lipid raft-associated protein ERLIN2 to cooperatively stabilize SREBP1 protein levels by inhibiting its ubiquitination. Furthermore, knockdown of either ERLIN2 or SREBP1 remarkably inhibits the proliferation and migration capabilities of NPC cells, synergizing with CLPTM1L depletion. Importantly, SREBP1 overexpression markedly restored the inhibitory effects mediated by CLPTM1L and ERLIN2 knockdown, underscoring SREBP1 as a critical mediator in CLPTM1L’s oncogenic role. These findings delineate a novel pathogenic mechanism in NPC, highlighting the KLF1/CLPTM1L/ERLIN2/SREBP1 regulatory cascade as a promising therapeutic target for NPC treatment.

## Introduction

Nasopharyngeal carcinoma (NPC) is a head and neck cancer originating from the epithelial cells of the nasopharynx. Its etiology is multifactorial and complex, involving Epstein-Barr virus (EBV) infection, exposure to environmental carcinogens, and personal lifestyle factors [1]. Accumulating studies have highlighted the critical role of genetic predisposition in NPC pathogenesis, as evidenced by distinct geographical and ethnic disparities in NPC incidence [2]. Genetic association studies have identified several susceptibility genes linked to increased NPC risk, such as HLA-A, HLA-B [3], MECOM [4], TNFRSF19 [5], CDKN2A/2B [6], and CLPTM1L/TERT [7]. However, despite these discoveries, the underlying mechanisms by which these genetic factors contribute to NPC tumorigenesis remain insufficiently understood.

CLPTM1L, also known as cisplatin resistance-related protein-9 (CRR9), was initially identified due to its elevated expression in cisplatin-resistant ovarian tumor cell lines [8]. Studies utilizing aggregated whole-genome association data have pinpointed the CLPTM1L locus as a significant region linked to increased risk of NPC [7, 9]. Moreover, the CLPTM1L genomic region has been associated with susceptibility to other cancers, including cervical cancer, melanoma, lung cancer, and pancreatic cancer [10–13], underscoring its broader role in cancer susceptibility. Recent research has also demonstrated the tumor-specific expression of CLPTM1L and its involvement in apoptosis resistance, particularly in lung and pancreatic cancers, where it enhances the expression of Bcl-xl, an anti-apoptotic protein [14, 15]. Additionally, CLPTM1L interacts with PI3K, promoting Ras-induced Akt phosphorylation and contributing to lung cancer development [16]. These findings suggest that CLPTM1L functions as an oncogene across multiple cancer types. Although CLPTM1L has been recognized as a susceptibility gene for NPC, its specific role in this context remains largely unexplored.

In this study, we systematically explored the functional role and underlying mechanisms of CLPTM1L in NPC. Our findings reveal that CLPTM1L is significantly upregulated in NPC and is associated with poor patient prognosis. Functional analyses confirmed the oncogenic potential of CLPTM1L in NPC cells. Mechanistically, we uncovered that CLPTM1L is transcriptionally regulated by KLF1. Furthermore, CLPTM1L interacts with ERLIN2 on the endoplasmic reticulum membrane, a critical interaction for stabilizing SREBP1, which regulates intracellular free fatty acid levels and promotes tumorigenesis. These findings delineate a novel mechanism by which CLPTM1L drives NPC development and progression, offering potential therapeutic targets for NPC treatment.

## Results

### High expression of CLPTM1L is associated with poor prognosis of NPC patients

**Fig. 1:**
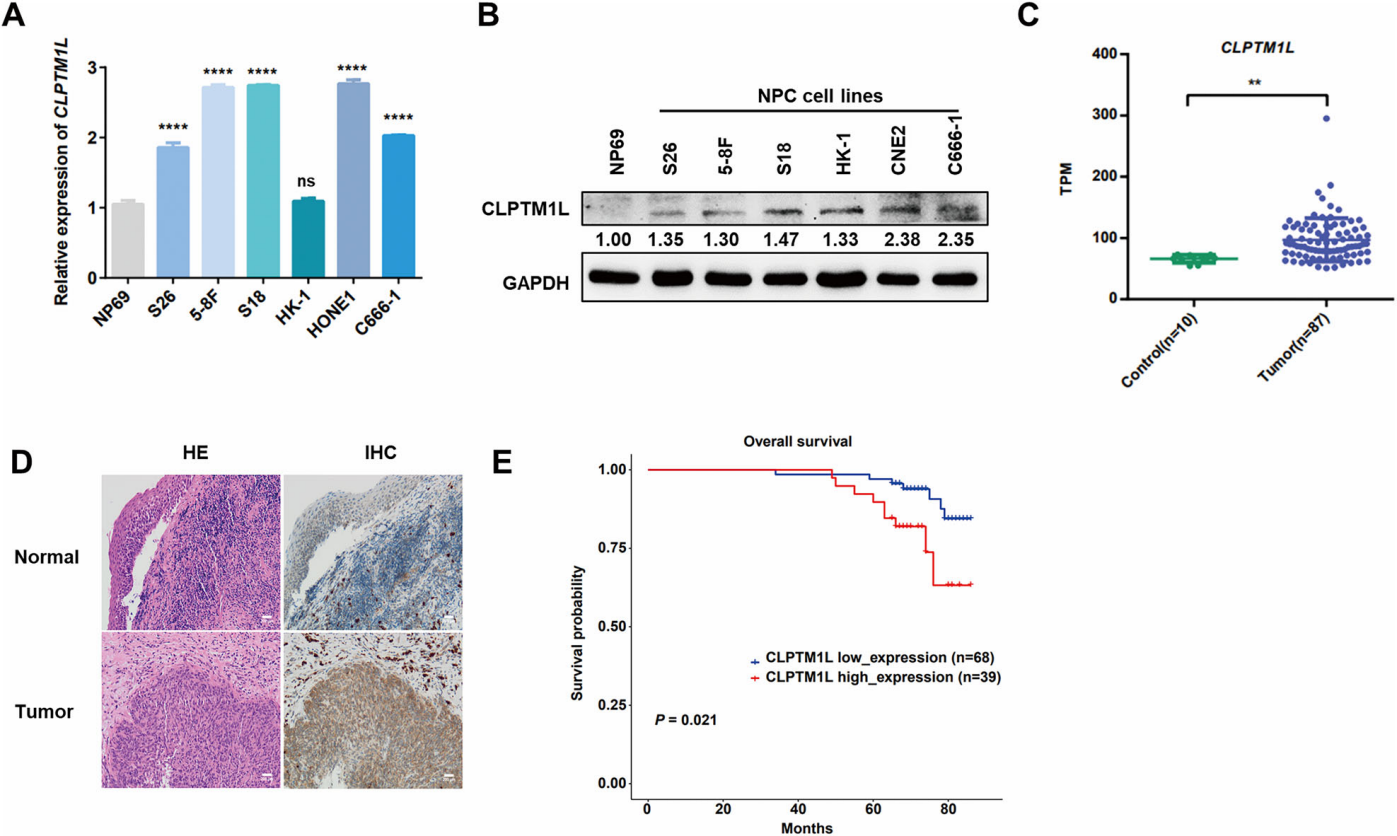
CLPTM1L is highly expressed in NPC and associated with poor prognosis. **A** RT-qPCR analysis measuring the mRNA expression of CLPTM1L in NPC cell lines and non-cancerous nasopharyngeal epithelial cells (NP69). **B** Western blotting assays showing the protein levels of CLPTM1L and GAPDH in cells described in (A). **C** Transcriptome analysis evaluating the mRNA expression of CLPTM1L in the NPC samples (n = 87) compared with control samples (n = 10). **D** Representative images of H&E and IHC staining with CLPTM1L antibody in NPC tumors and normal nasopharyngeal tissues. Scale bars, 100 μm. **E** Kaplan–Meier survival curves of overall survival (OS) in NPC patients (n = 107) with high or low CLPTM1L expression. Statistical analysis is performed by Student’s t-test for (**C**) and log-rank test for (**E**), with data presented as the mean ± SD. *P < 0.05; **P < 0.01; ***P < 0.001; ****P < 0.0001.

To explore the clinical significance of CLPTM1L in NPC, we first evaluated its expression in NPC cell lines and tissues. Our analysis revealed a remarkable upregulation of CLPTM1L at both mRNA and protein levels in NPC cell lines compared to the nasopharyngeal epithelial cell line NP69 (Fig. 1A, B). This elevated expression pattern was also observed in NPC biopsy samples, where CLPTM1L levels were markedly higher than in control tissues from patients with rhinitis, as revealed by transcriptome analysis (Fig. 1C). Moreover, immunohistochemical analysis with CLPTM1L antibodies demonstrated that CLPTM1L predominantly localized in the cytoplasm of tumor cells, with minimal expression in adjacent normal tissues (Fig. 1D). Kaplan–Meier survival analysis further revealed that NPC patients with high CLPTM1L expression exhibited worse prognosis compared to those with lower expression levels (Fig. 1E), indicating its important role in NPC progression.

### CLPTM1L functions as an oncogene in NPC

**Fig. 2:**
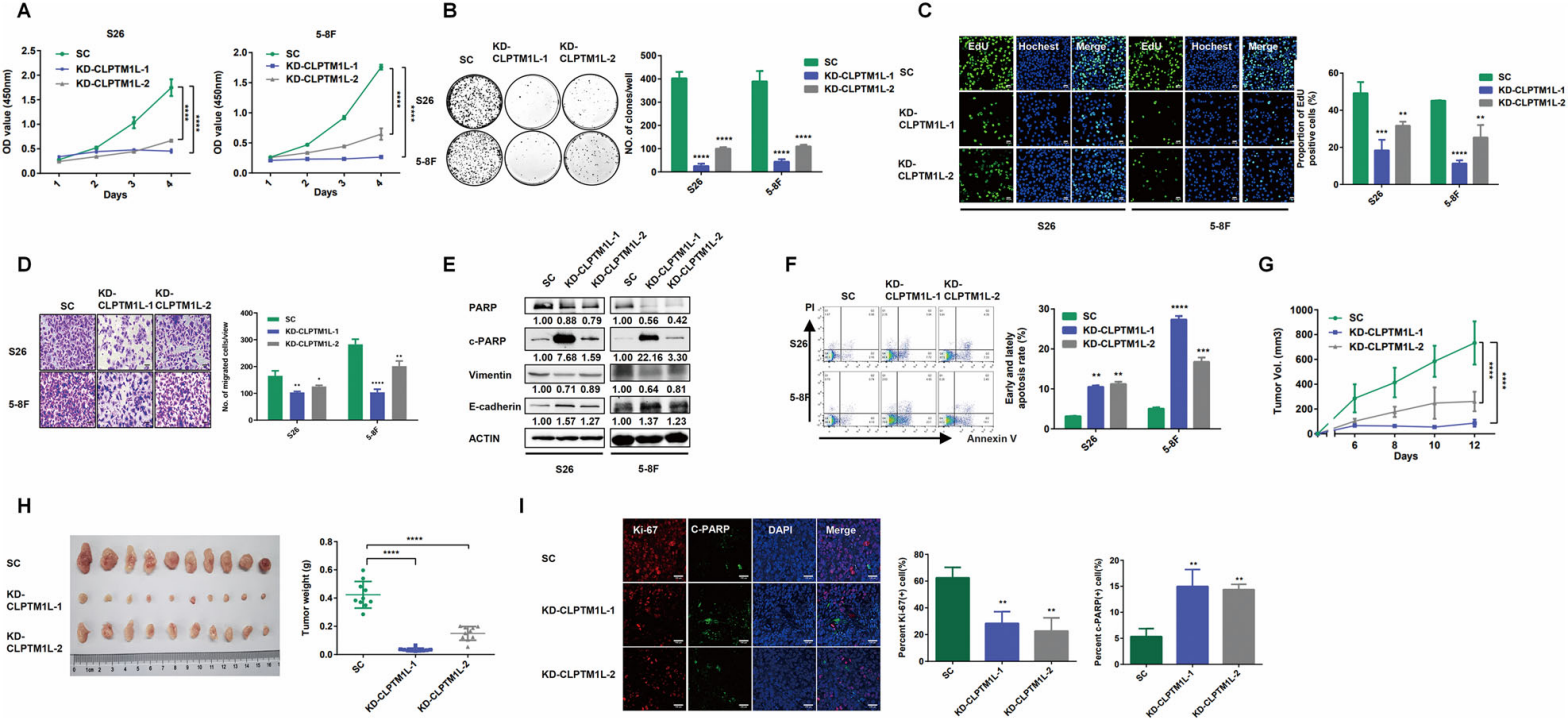
CLPTM1L functions as an oncogene in NPC. **A** CCK8 assay measuring the proliferation abilities of S26 and 5–8 F cells transfected with CLPTM1L siRNAs (KD-CLPTM1L-1/2) or control siRNA (SC). Differences between groups were analyzed by two-way ANOVA. **B** Colony formation assay is conducted with cells described in (**A**), with statistics presented at the right. **C** Representative images of EdU staining with cells described in (**A**). The statistical analysis is shown at the right. **D** Transwell assay showing the migration abilities of cells described in (**A**), alongside with statistics at the right. **E** Western blotting analysis demonstrating the protein levels of PARP, cleaved-PARP, Vimentin, and E-cadherin in CLPTM1L-knockdown cells described in (**A**). ACTIN is used as an internal control. **F** Flow cytometry analysis with FITC Annexin V and PI in cells described in (**A**). **G** Growth curve of xenograft tumors derived from S26 cells stably expressing CLPTM1L shRNAs (KD-CLPTM1L-1/2) or control shRNA (SC). **H** The tumor size (left) and weight (right) are shown. **I** Representative images of IF staining against Ki-67 and c-PARP antibodies in tumors described in (**H**). Statistical analysis is performed by one-way ANOVA test and data are presented as the mean ± SD. *P < 0.05; **P < 0.01; ***P < 0.001; ****P < 0.0001. Scale bars, 100 μm.

To elucidate the functional role of CLPTM1L in NPC development, we established NPC cell lines (S26 and 5–8 F) with knockdown of CLPTM1L expression using two independent siRNAs (Fig. S1A). Notably, CLPTM1L knockdown substantially inhibited NPC cell proliferation, as evidenced by decreased cell growth rate and colony-forming capacity, along with a lower percentage of EdU (+) cells, a well-known proliferative marker, compared with control cells (Fig. 2A-C). Transwell and wound healing assays further demonstrated that CLPTM1L knockdown suppressed cell migration and invasion (Fig. 2D, Fig. S1B, C), corroborated by a decrease in the mesenchymal marker Vimentin and an increase in the epithelial marker E-cadherin, indicating reduced cellular motility (Fig. 2E). Additionally, flow cytometry analysis with Annexin V revealed increased apoptosis in NPC cells with CLPTM1L depletion, further supported by elevated expression of the apoptosis marker cleaved-PARP (Fig. 2E, F). To further verify the oncogenic potential of CLPTM1L, we generated NPC cells overexpressing CLPTM1L (Fig. S2A). We observed enhanced the proliferation, migration and invasion capacities in NPC cells with CLPTM1L overexpression (Fig. S2B–I), in stark contrast to the inhibitory effects observed following CLPTM1L knockdown.

We next evaluated CLPTM1L’s role in vivo using xenograft models by subcutaneously implanting CLPTM1L-knockdown S26 or control cells into nude mice. Tumor growth was monitored over time, revealing a significantly slower growth rate in the CLPTM1L-knockdown group compared to controls (Fig. 2G). This reduction was further substantiated by a marked decrease in tumor volume and weight in the CLPTM1L-knockdown group (Fig. 2H). Immunofluorescence staining of tumor sections demonstrated decreased Ki-67 (+) and increased cleaved-PARP (+) cells, indicating reduced cell proliferation and increased apoptosis in CLPTM1L-depleted tumors (Fig. 2I). Together, these findings strongly suggest that CLPTM1L is essential for the proliferation, migration and tumorigenesis of NPC cells, emphasizing its critical function in NPC progression.

### KLF1 positively regulates CLPTM1L transcription

**Fig. 3:**
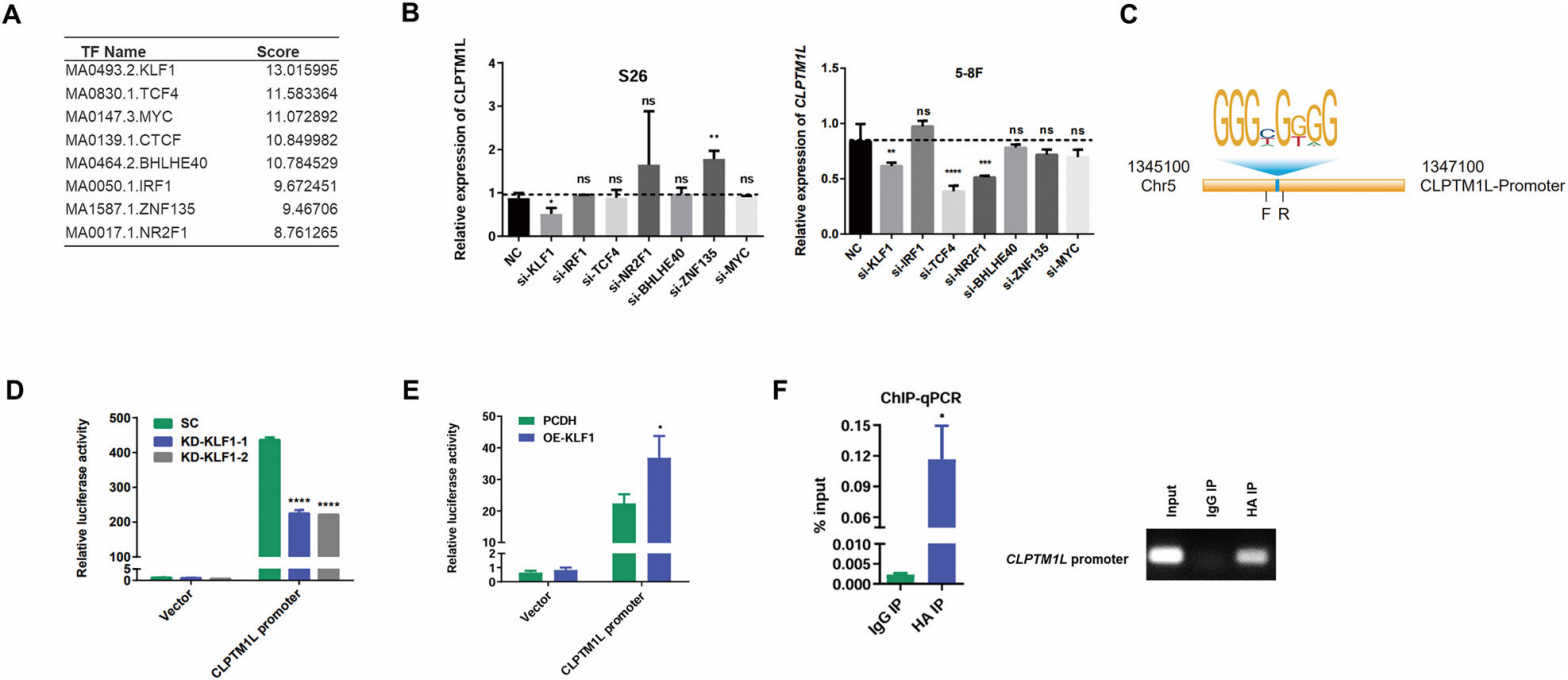
KLF1 promotes the transcription of CLPTM1L. **A** Prediction of candidate transcription factors for CLPTM1L with UCSC and JASPAR database. **B** RT-qPCR analysis measuring the mRNA expression of CLPTM1L in S26 and 5–8 F cells following knockdown of candidate transcription factors from (**A**). **C** Schematic diagram of the CLPTM1L promoter. The KLF1-binding motif is represented by the sequence GGGC/TGG/TGG. Primer sequences used for amplification are indicated as follows: F (Forward primer) and R (Reverse primer). **D** Luciferase reporter assay in S26 cells co-transfected with pGL3 plasmid containing the CLPTM1L promoter region or control vector, alongside with KLF1 siRNA or control siRNA. **E** Luciferase reporter assay in S26 cells co-transfected with pGL3 plasmid containing the CLPTM1L promoter region or control vector, alongside with KLF1 overexpressing plasmids or control vector. **F** ChIP-PCR analysis with anti-HA antibody revealing KLF1’s interaction with the CLPTM1L promoter. Statistical analysis is performed by one-way ANOVA test and Student’s t-test for (**E**, **F**), with data presented as the mean ± SD. *P < 0.05; **P < 0.01; ***P < 0.001; ****P < 0.0001.

To identify upstream regulators of CLPTM1L, we utilized the UCSC and JASPAR databases to predict transcription factors potentially binding to the CLPTM1L promoter [17, 18]. Specially, eight candidate transcription factors were identified, with higher scores indicating more reliable predictions (Fig. 3A). We subsequently conducted an siRNA screen targeting these candidates, which verified that KLF1 regulates CLPTM1L expression (Fig. 3B). To further investigate the regulatory impact of KLF1 on CLPTM1L transcription, we conducted dual-luciferase assays by inserting the promoter region of CLPTM1L (~2000bp upstream from the transcription start site), including the KLF1 binding motif, into the pGL3 vector (Fig. 3C). We observed that KLF1 knockdown significantly reduced the transcription activity of CLPTM1L in NPC cells (Fig. 3D), whereas its overexpression exhibited promoting effects (Fig. 3E). Furthermore, chromatin immunoprecipitation (ChIP) assay followed by PCR confirmed the direct binding of KLF1 to the CLPTM1L promoter (Fig. 3F), elucidating KLF’s role in upregulating CLPTM1L expression in NPC.

### CLPTM1L regulates the fatty acid metabolic pathway through SREBP1

**Fig. 4:**
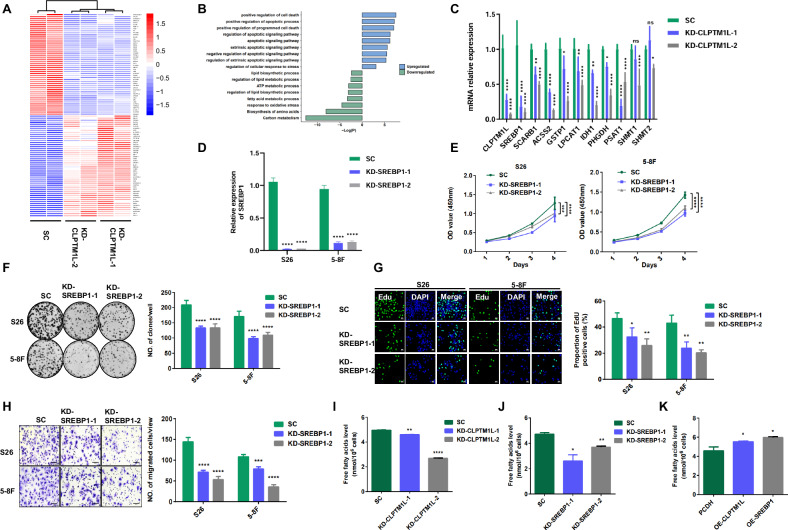
CLPTM1L positively regulates the fatty acid metabolic pathway through SREBP1. **A** Heat map depicting the global mRNA expression profile 5–8 F cells transfected with CLPTM1L siRNA or control siRNA. **B** Gene Ontology analysis of differentially expressed genes (DEGs) from (**A**). **C** RT-qPCR analysis evaluating the mRNA levels of pathway-related genes in cells described in (**A**). **D** RT-qPCR analyses showing the knockdown efficiency of SREBP1 in NPC cells transfected with indicated siRNAs (KD-1/2) or control siRNA (SC). **E** CCK8 assay measuring the proliferation of S26 and 5-8 F cells transfected with SREBP1 siRNA or control siRNA. **F** Colony formation assay is conducted with cells described in (**D**), with statistics presented at the right. **G** Representative images of EdU staining with cells described in (**D**). **H** Transwell assay showing the migration abilities of cells described in (D), alongside with statistics at the right. **I, J** Quantification of cellular free fatty acids in S26 cells with CLPTM1L (**I**) or SREBP1 knockdown (**J**). **K** Quantification of cellular free fatty acids levels in S26 cells overexpressing CLPTM1L or SREBP1. Statistical analysis is performed by one-way ANOVA test and data are presented as the mean ± SD. *P < 0.05; **P < 0.01; ***P < 0.001; ****P < 0.0001.

We next elucidated the oncogenic mechanisms of CLPTM1L in NPC by conducting transcriptome sequencing of 5-8 F cells following CLPTM1L knockdown. This analysis revealed 99 differentially expressed genes, with 49 genes downregulated and 50 genes upregulated upon CLPTM1L depletion (Fig. 4A). Gene Ontology (GO) enrichment analysis indicated that the upregulated genes were predominantly involved in apoptotic signaling pathways (Fig. 4B), aligning with the increased apoptosis in CLPTM1L-knockdown cells. In contrast, the downregulated were primarily associated with lipid metabolism, energy metabolism, oxidative stress, and amino acid biosynthesis (Fig. 4B), indicating a critical role for CLPTM1L in regulating these metabolic pathways. We further validated decreased expression of key genes related to these pathways, particularly various metabolic processes, in CLPTM1L-knockdown cells (Fig. 4C). To investigate whether CLPTM1L facilitates NPC progression by regulating these metabolic genes, we performed siRNA-mediated knockdown of several critical metabolic genes, including SREBP1, ACSS2, LPCAT1, and SCARB1, in NPC cells (Figs. 4D and S3A). Functional assays revealed that knockdown of each of these genes significantly impaired NPC cell proliferation and migration (Figs. 4E–H and S3B–D), underscoring the broader role of CLPTM1L in modulating NPC progression.

Among the metabolic genes examined, SREBP1 exhibited the most significant downregulation upon CLPTM1L knockdown, positioning it as the primary target for further investigation. Previous studies have highlighted SREBP1’s crucial role in regulating cholesterol and fatty acid metabolism [19], implicating a potential involvement of CLPTM1L in fatty acid biosynthesis, which is essential for tumor cell proliferation and energy demands. Strikingly, CLPTM1L inhibition led to a remarkable reduction in intracellular free fatty acid levels in S26 cells (Fig. 4I), consistent with the effects mediated by SREBP1 knockdown (Fig. 4J). Conversely, overexpression of CLPTM1L or SREBP1 notably increased intracellular free fatty acids levels (Fig. 4K). These findings suggest that CLPTM1L regulates the fatty acid metabolic pathway to promote NPC progression, particularly through modulation of SREBP1.

### CLPTM1L directly interacts with ERLIN2 in NPC

**Fig. 5:**
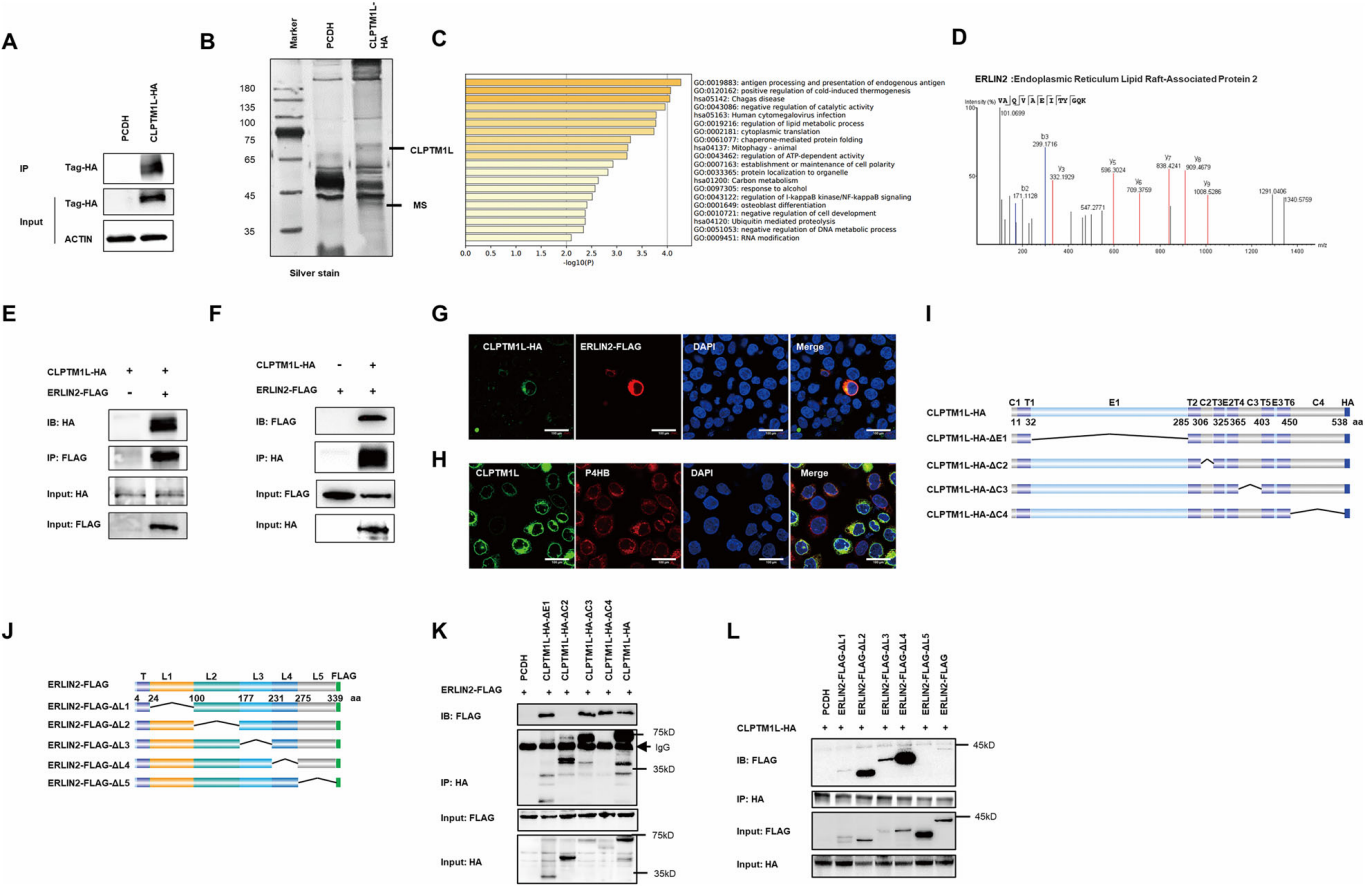
CLPTM1L directly interacts with ERLIN2 in NPC cells. **A** Immunoprecipitation (IP) assay in S26 cells expressing CLPTM1L-HA or control vectors, validated by the western blotting assay. **B** Identification of CLPTM1L-interacting proteins. Proteins extracted from S26 cells expressing CLPTM1L-HA or control vectors are incubated with Protein A/G Magnetic Beads conjugated with anti-HA antibody, subjected to SDS-PAGE and visualized by silver staining. The band indicated is subjected to mass spectrometry. **C** Gene Ontology enrichment analysis of candidate proteins identified via mass spectrometry. **D** IP-MS identifying the ERLIN2 peptides in CLPTM1L-interacted proteins. **E**, **F** Co-IP assays showing ERLIN2-Flag pulled down by CLPTM1L-HA (E) and CLPTM1L-HA pulled down by ERLIN2-Flag (F). **G** Immunofluorescent staining showing the subcellular localization of CLPTM1L (green) and ERLIN2 (red) in S26 cells. **H** Immunofluorescent staining showing the subcellular localization of CLPTM1L (green) and endoplasmic reticulum marker P4HB (red) in S26 cells. Scale bars, 100 μm. **I** Schematic diagram of the domain structure of wild-type CLPTM1L-HA and its mutants constructs. T, transmembrane, presented as purple box; E, extracellular domain, presented as blue box; C, cytoplasmic domain, presented as gray box. The dark blue box indicates the HA tag and the numbers present the amino acid number. **J** Schematic diagram of the domain structure of wild-type ERLIN2-FLAG and its deletion mutants. T, transmembrane, presented as purple box; L, Lumenal, which is divided into five regions (L1–L5) presented as different colors. The Flag tag is indicated as the green box and the numbers present the amino acid number. **K** Co-IP assays in 293T cells co-transfected with wild-type ERLIN2-FLAG and either wild-type CLPTMIL-HA or the indicated deletion mutants. Immunoprecipitations were performed using anti-HA antibodies. **L** Co-IP assays in 293T cells co-transfected with wild-type CLPTMIL-HA and either wild-type ERLIN2-FLAG or the indicated deletion mutants. Immunoprecipitations were performed using anti-HA antibodies. The “+” symbol indicates the presence of indicated construct in the experiment. IP immunoprecipitate, IB immunoblot.

To delineate how CLPTM1L regulates SREBP1 expression in NPC, we conducted immunoprecipitation followed with mass spectrum (IP-MS) to explore the interacting proteins of CLPTM1L (Fig. 5A, B). The analysis identified 63 candidate proteins, predominantly enriched in lipid metabolic pathways according to GO analysis (Fig. 5C). Among these, ERLIN2, a lipid raft-associated protein, was highlighted as one of the top ten proteins (Fig. 5D). Considering ERLIN2’s role in cholesterol homeostasis and lipid metabolism [20], we propose that CLPTM1L may influence lipid metabolism through interacting with ERLIN2. Indeed, both forward and reverse immunoprecipitation assays verified the direct interaction between CLPTM1L and ERLIN2 in NPC cells (Fig. 5E, F). Moreover, immunofluorescence assays demonstrated clear co-localization of these two proteins (Fig. 5G). Given the well-established endoplasmic reticulum (ER) localization of ERLIN2 [21], we further revealed co-staining of CLPTM1L with the ER marker P4HB (Fig. 5H), indicating a collaborative role of these proteins in modulating lipid metabolic pathways in NPC cells.

To pinpoint the specific domains that mediate the CLPTM1L-ERLIN2 interaction, we analyzed both proteins using the UniProt database (https://www.uniprot.org). Our analysis revealed that CLPTM1L consists of multiple extracellular and cytoplasmic topological domains, as well as transmembrane regions (Fig. 5I), whereas ERLIN2 contains a large lumenal domain and a helical transmembrane domain (Fig. 5J). To further investigate this interaction, we generated a series of domain-deletion mutant constructs. For CLPTM1L, we systematically deleted each of the four major domains, including the first extracellular domain (E1), and the second to the fourth cytoplasmic domain (C2, C3 and C4; Fig. 5I). For ERLIN2, we subdivided its large lumenal domain into five regions and deleted each portion individually (Fig. 5J). Co-immunoprecipitation (Co-IP) assays demonstrated that deletion of the second cytoplasmic domain in CLPTM1L (CLPTM1L-HA-ΔC2) abolished its interaction with ERLIN2 (Fig. 5K). Similarly, the ERLIN2 mutant lacking its C-terminal region (ERLIN2-FLAG-ΔL5) failed to interact with CLPTM1L (Fig. 5L). These findings indicate that the second cytoplasmic domain of CLPTM1L and the C-terminal region of ERLIN2 are essential for their interaction.

### CLPTM1L and ERLIN2 cooperate to stabilize SREBP1 by inhibiting its ubiquitination

**Fig. 6:**
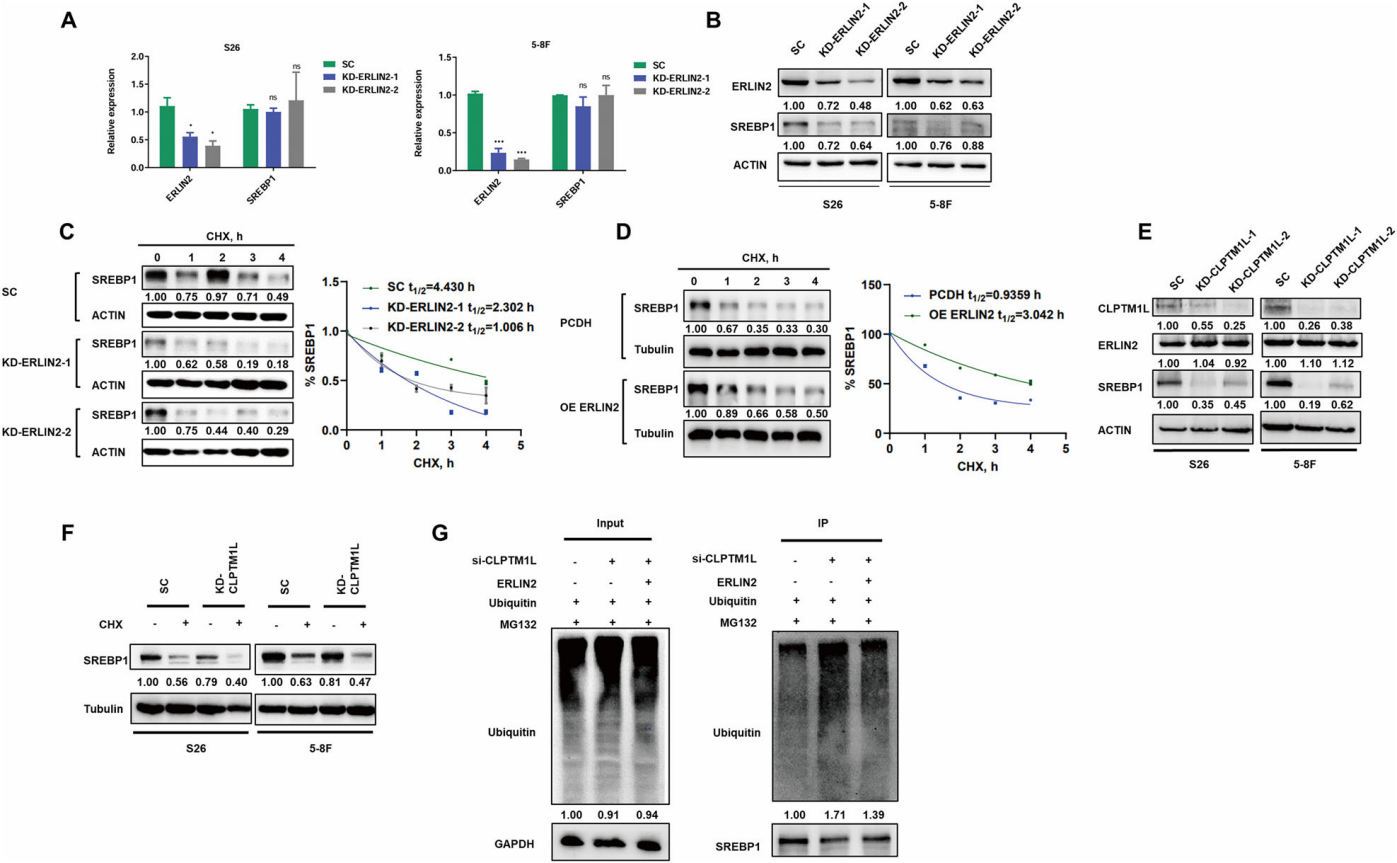
CLPTM1L and ERLIN2 jointly inhibit SREBP1 degradation by reducing its ubiquitination. RT-qPCR (**A**) and western blot (**B**) analysis of SREBP1 mRNA expression and protein level in S26 and 5-8 F cells with CLPTM1L knockdown. ACTIN is used as an internal control. Western blot analysis shows the protein stabilities of SREBP1 in ERLIN2 knockdown (**C**) or overexpression (**D**) S26 cells treated with cycloheximide (CHX) for different time points. SREBP1 signals were quantified, normalized to ACTIN (**C**, right) or Tubulin (**D**, right). **E** Western blot analysis showing the protein levels of CLPTM1L, ERLIN2 and SREBP1 in CLPTM1L knockdown NPC cells, with ACTIN as an internal control. **F** Western blot analysis of SREBP1 levels in CLPTM1L-knockdown NPC cells treated with or without CHX for 4 h. **G** Western blot analysis of SREBP1 ubiquitination in CLPTM1L-knockdown S26 cells transfected with ERLIN2 and Ubiquitin-HA plasmids, and subsequently incubated with MG132 for 6 h.

We next investigated whether ERLIN2 regulates SREBP1 expression. Notably, ERLIN2 knockdown reduced SREBP1 protein levels without affecting its mRNA levels (Fig. 6A, B), suggesting that ERLIN2 modulates SREBP1 stability through a post-transcriptional mechanism. To further explore this, we treated ERLIN2-knockdown S26 cells with cycloheximide, a protein synthesis inhibitor, and monitored SREBP1 protein degradation over time. The results showed that SREBP1 protein degradation was significantly accelerated in ERLIN2-knockdown cells compared to controls (Fig. 6C). Conversely, ERLIN2 overexpression in S26 cells markedly slowed the degradation of SREBP1 protein (Fig. 6D), supporting the hypothesis that ERLIN2 maintains the protein stability of SREBP1. To determine if ERLIN2 stabilizes SREBP1 through preventing proteasome-mediated degradation, we employed MG132, a proteasome inhibitor, to treat ERLIN2-knockdown cells, in combination with cycloheximide. Remarkably, the reduction in intracellular SREBP1 protein levels post-cycloheximide treatment was almost restored with MG132 treatment (Fig. S4A). Furthermore, immunoprecipitation assays confirmed that ERLIN2 knockdown led to increased ubiquitination levels of SREBP1 (Fig. S4B). These findings collectively suggest that ERLIN2 knockdown promotes the proteasomal degradation of SREBP1 through enhancing its ubiquitination.

To further clarify CLPTM1L’s role within the regulatory network involving ERLIN2 and SREBP1, we first examined the effects of CLPTM1L on ERLIN2 in NPC cells. Strikingly, CLPTM1L knockdown led to a significant decrease of SREBP1 protein levels without altering ERLIN2 levels (Fig. 6E), consistent with findings from CLPTM1L-knockdown tumors in vivo (Fig. S4C). We further investigated CLPTM1L’s necessity for SREBP1 stability by treating CLPTM1L-knockdown NPC cells with cycloheximide and observed an accelerated degradation of SREBP1 protein (Fig. 6F). Additionally, immunoprecipitation assays demonstrated increased SREBP1 ubiquitination following CLPTM1L knockdown (Fig. S4D). To further confirm the cooperative regulation of SREBP1 by CLPTM1L and ERLIN2, rescue experiments were conducted by overexpressing ERLIN2 in CLPTM1L-knockdown cells. Supportively, ERLIN2 overexpression partially restored SREBP1 protein levels (Fig. S4E). Furthermore, the increased ubiquitination levels of SREBP1 caused by CLPTM1L knockdown were significantly reversed by ERLIN2 overexpression (Fig. 6G), highlighting the collaborative role of CLPTM1L and ERLIN2 in stabilizing SREBP1 by regulating its ubiquitination.

### SREBP1 mediates the tumorigenic roles of CLPTM1L and ERLIN2

**Fig. 7:**
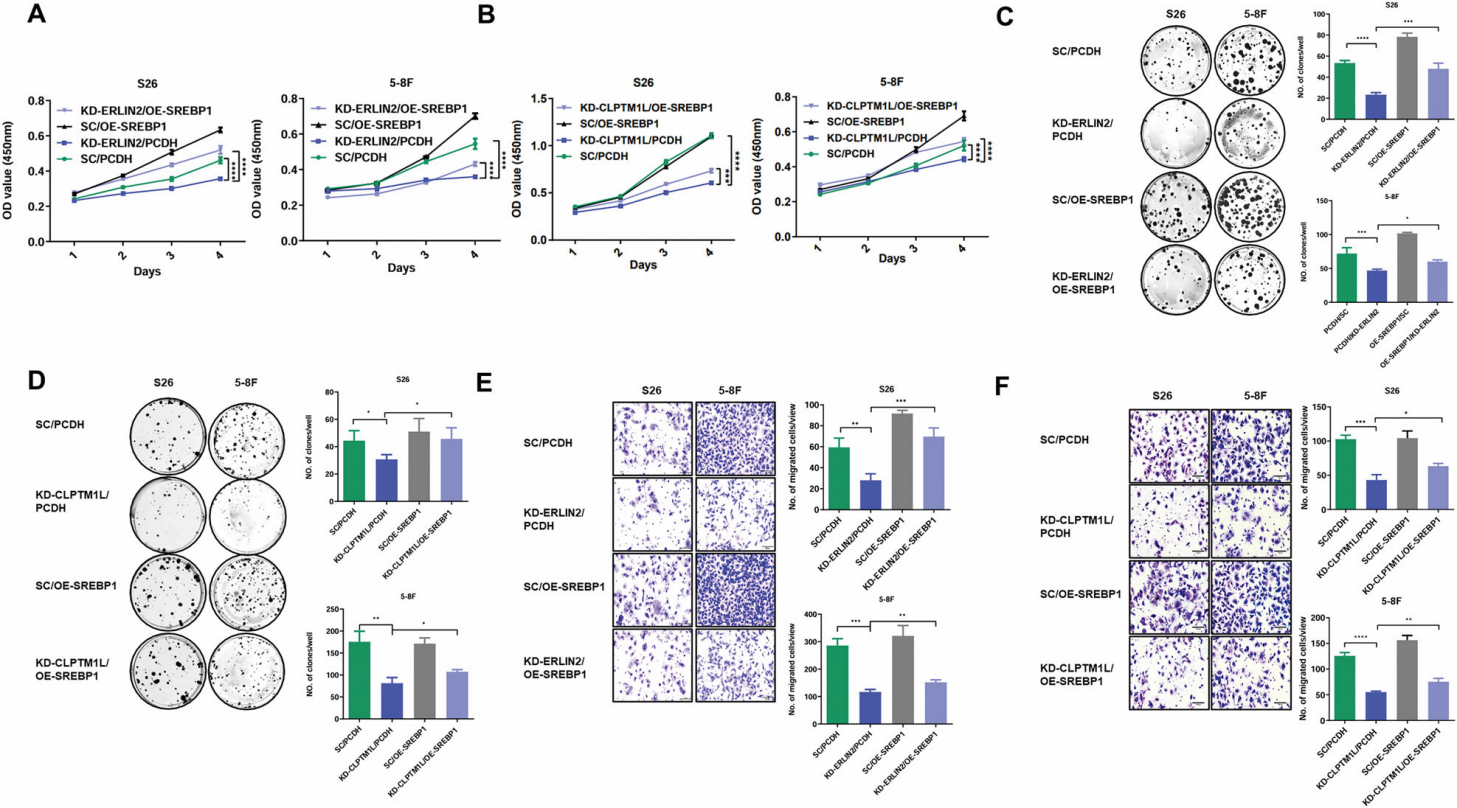
SREBP1 rescues phenotypes caused by knockdown of CLPTM1L or ERLIN2. **A** CCK8 assay in S26 and 5–8 F cells expressing ERLIN2 shRNA or control shRNA, and simultaneously infected with SREBP1 overexpression or control lentiviruses. **B** CCK8 assay in S26 and 5-8 F cells expressing CLPTM1L shRNA or control shRNA, and simultaneously infected with SREBP1 overexpression or control lentiviruses. **C**, **D** Colony formation assay with cells described in (**A**, **B**). **E**, **F** Transwell assay with cells described in (**A**, **B**). Statistical analysis is performed by one-way ANOVA test and data are presented as the mean ± SD. *P < 0.05; **P < 0.01; ***P < 0.001; ****P < 0.0001. Scale bars, 100 μm.

Subsequently, we explored the functional role of ERLIN2 in NPC using siRNA-mediated knockdown. Notably, ERLIN2 inhibition resulted in a significant reduction in cell proliferation and migration capabilities in NPC cells compared to control cells (Fig. S5A–C), consistent with the oncogenic roles of CLPTM1L and SREBP1. To further elucidate the functional link between CLPTM1L, ERLIN2, and SREBP1 in NPC, we conducted a series of rescue experiments (Fig. S6A, B). We observed that knockdown of either CLPTM1L or ERLIN2 substantially diminished the proliferation ability of NPC cells; however, these inhibitory effects were successfully restored with SREBP1 overexpression (Fig. 7A–D). Similarly, transwell assays revealed that SREBP1 overexpression significantly reversed the reduction in cell migration caused by CLPTM1L or ERLIN2 knockdown (Fig. 7E, F). In contrast, overexpression of aforementioned LPCAT1, ACSS2, or SCARB1 failed to rescue the proliferation and migration defects induced by CLPTM1L knockdown (Fig. S7A–D). These findings further underscore the central role of SREBP1 among CLPTM1L’s target genes in mediating its oncogenic effects in NPC.

## Discussion

Previous studies have identified CLPTM1L as a susceptibility gene associated increased risk of NPC [7, 9]. Our study extends these findings by establishing CLPTM1L as an oncogene in NPC. We demonstrate that elevated CLPTM1L expression correlates with poor prognostic outcomes. Both in vitro and in vivo experiments show that CLPTM1L knockdown markedly reduces the proliferation, migration, invasion and tumorigenesis of NPC cells, whereas its overexpression demonstrates opposite promoting effects. This is consistent with findings from other malignancies, such as lung and pancreatic cancers, where CLPTM1L has been implicated in promoting tumor growth through resistance to apoptosis and activation of the PI3K/Akt pathway [15, 16]. These results underscore CLPTM1L’s oncogenic role and suggest it may serve as a therapeutic target in various cancers, including NPC.

We further reveal that CLPTM1L is transcriptionally regulated by KLF1, a member of the Krüppel-like factor (KLF) family, through a direct binding to CLPTM1L promoter. As a DNA-binding transcriptional regulator, KLF1 plays critical roles in various cellular processes, including proliferation [22], differentiation [23], migration [24], inflammation [25], and pluripotency [26]. Additionally, previous studies have shown that transcription factor CTCF binds to CLPTM1L promoter and regulates its expression in breast cancer cell [27]. These findings suggest that the regulatory mechanisms of CLPTM1L transcription are various among different cancer contexts, underscoring its critical role in promoting tumorigenesis.

Our study demonstrates that CLPTM1L knockdown leads to the downregulation of numerous genes involved in various metabolic pathways, with a significant impact on fatty acid metabolism. Functional assays identified several key metabolic genes as critical regulators of NPC cell function, highlighting the extensive role of CLPTM1L in modulating NPC progression. Furthermore, we reveal that CLPTM1L upregulates SREBP1 expression, increasing intracellular free fatty acid levels in NPC cells. This observation aligns with SREBP1’s role as a well-known regulator of lipid synthesis, controlling the expression of genes such as acetyl-CoA carboxylase, fatty acid synthase, and stearoyl-CoA desaturase, all of which are critical for cancer-related metabolic reprogramming [28]. These findings emphasize the pivotal role of CLPTM1L not only in its classical regulation of apoptosis pathways but also in driving metabolic reprogramming to facilitate NPC progression through modulation of SREBP1. Supporting this, previous studies have reported the upregulation of SREBP1 in various cancers [29, 30]. Specifically in NPC, long non-coding RNA LINC02570 and Epstein-Barr virus-encoded LMP1 upregulate SREBP1, thereby promoting NPC progression [31, 32].

We further elucidate the mechanisms by which CLPTM1L interacts with the C-terminal of ERLIN2 via its second cytoplasmic domain, thereby stabilizing SREBP1 protein levels via the ubiquitin-proteasome system. ERLIN2, a lipid raft-associated protein, has been previously implicated in cholesterol homeostasis through endoplasmic reticulum-associated protein degradation (ERAD) [33, 34]. While ERLIN2 is conventionally known for promoting protein degradation, including facilitating the ubiquitination and degradation of activated inositol trisphosphate receptors [21], our findings reveal a distinct function. In cooperation with CLPTM1L in the endoplasmic reticulum, ERLIN2 stabilizes SREBP1 by preventing its ubiquitination. This context-specific role suggests that ERLIN2 may have dual functions depending on its interacting partners and the cellular environment, shifting from a facilitator of degradation to a stabilizer of key metabolic regulators, such as SREBP1. Furthermore, knockdown of ERLIN2 significantly inhibits NPC cell proliferation and migration, with these suppressive effects being notably reversed by SREBP1 overexpression. These findings underscore the critical role of ERLIN2/SREBP1 axis in CLPTM1L-mediated tumorigenesis in NPC.

In summary, our study delineates a novel mechanism by which CLPTM1L in modulates fatty acid metabolism to drive NPC progression. Mechanistically, KLF1 facilitates CLPTM1L transcription through binding to its promoter. The upregulated CLPTM1L further directly interacts with ERLIN2 to stabilize SREBP1 by inhibiting its ubiquitination, thereby promoting enhanced fatty acid metabolism in NPC cells. These findings offer critical insights into NPC pathophysiology and provide potential therapeutic targets to disrupt metabolic reprogramming in this malignancy.

## Materials and methods

### Clinical sample collection

NPC tissues and control tissues were obtained from patients diagnosed between January 4, 2011 and September 30, 2013, at Sun Yat-sen University Cancer Center (SYSUCC), Guangzhou, China. Fresh tissue samples were promptly frozen in liquid nitrogen and stored at -80 °C until further use for RNA extraction. Additionally, paraffin-embedded biopsy specimens were sectioned for immunohistochemistry analysis. Histopathological diagnoses were independently confirmed by at least two pathologists according to the WHO classification criteria. Clinical staging of NPC patients was performed following the 7th edition of the UICC and AJCC staging system. Written informed consent was obtained from all participants, and the study protocol was approved by the Institutional Review Board of SYSUCC.

### Cell culture

Human embryonic kidney 293T (HEK293T) cells were obtained from the Cell Bank of Type Culture Collection of the Chinese Academy of Sciences (Shanghai, China). Human NPC cell lines (S26 and 5–8 F) were generously provided by Professor Chaonan Qian at SYSUCC (Guangzhou, China). All cell lines were cultured in Dulbecco’s modified Eagle’s medium (DMEM, Gibco, NY, USA,C11995500BT), supplemented with 10% fetal bovine serum (EXCELL, FSP500) and 1% Penicillin–Streptomycin (Gibco, NY, USA, 15140122). Regular screening for mycoplasma contamination was performed, and all cell lines tested negative for mycoplasma.

### siRNAs, plasmids construction and lentivirus packaging

Gene silencing was conducted using siRNA duplexes (GenePharma, Shanghai, China) targeting CLPTM1L, ERLIN2, SREBP1, and KLF1. CLPTM1L knockdown in NPC cells was generated using the plasmid pLKO.1 vector expressing the shRNA against *CLPTM1L*. The same plasmid expressing shRNA targeting luciferase was used as control. Overexpression studies of these genes were performed using pCDH plasmids containing the respective cDNAs. To produce lentiviral particles, the lentiviral expression plasmid, along with the packaging plasmid psPAX2 and the envelope plasmid pMD2.G, were co-transfected into HEK-293T cells using Lipofectamine 2000 (Invitrogen) following the manufacturer’s instructions. After 48 h, the produced lentiviruses were used to infect NPC cells, which were subsequently selected with puromycin (2 μg/mL) for 3 days.

### RNA isolation and qPCR with reverse transcription

Total RNA was isolated with Trizol reagent (Invitrogen, California, USA, 15596018CN) following the manufacturer’s instructions. RNA concentration and purity were measured with a NanoDrop 2000 Spectrophotometer (Thermo Fisher Scientific, Waltham, MA, USA). cDNA was synthesized using the EasyScript® One-Step gDNA Removal and cDNA Synthesis SuperMix (TransGen Biotech, AE311-02). Quantitative real-time PCR (qPCR) was carried out with TB Green Premix Ex Taq (TAKARA, Tokyo, Japan, RR420A) on a Bio-Rad CFX96 Touch Real-Time PCR Detection System to evaluate the relative expression levels of target genes, normalized to the housekeeping gene β-actin. Specific primers were listed in Supplementary Table 3.

### Xenograft experiments

Six- to eight-week-old male BALB/C-nude mice were housed under specific pathogen-free (SPF) conditions. The mice were randomly assigned into groups, with each group consisting of 5 individuals. A total of 1×10^6^ cells (S26-SC, S26-KD CLPTM1L1-1, and S26-KD CLPTM1L1-2) were resuspended in Matrigel and subcutaneously injected into the flanks of the mice in a total volume of 100 μL per injection site. Tumor growth was monitored every other day by measuring tumor dimensions with calipers, and tumor volume was calculated using the formula V = 1/2a²b, where “a” denotes the shortest diameter and “b” the longest diameter. After 4 weeks, the mice were sacrificed, and tumors were excised and weighed for subsequent analysis. All animal care and experimental procedures in this study were carried out in accordance with institutional ethical guidelines and approved by the Institutional Review Board of SYSUCC.

### Western blotting

Cells were harvested using lysis buffer (Cell Signaling Technology, Danvers, USA, 9803) supplemented with protease inhibitors (Beyotime, P1005). The lysates were clarified by centrifugation and subsequently mixed with loading buffer, followed by denaturation at 100 °C for 10 min. Proteins were separated by SDS-PAGE and transferred onto polyvinylidene fluoride (PVDF) membranes (Merck Millipore, Billerica, MA, USA). Subsequently, the membranes were blocked with 5% bovine serum albumin (BSA) in TBS-T (Tris-buffered saline containing 0.05% Tween-20) for 1 h at room temperature. Membranes were then incubated overnight at 4 °C with primary antibodies, which were diluted in 5% BSA in TBS-T according to the manufacturer’s recommendations. The following day, the membranes were washed with TBS-T and incubated for 1 h at room temperature with horseradish peroxidase (HRP)-linked secondary antibodies. The protein bands were visualized using the Bio-Rad ChemiDoc Touch Imaging System (Hercules, USA). All antibodies were listed in Supplementary Table 4.

### Co-Immunoprecipitation (Co-IP)

Cells were washed with phosphate-buffered saline (PBS), and lysed on ice for 30 minutes in lysis buffer (Cell Signaling Technology, Danvers, USA, 9803) supplemented with protease inhibitors (Beyotime, Shanghai, China, P1005). The lysates were clarified by centrifugation at 14,000 × *g* for 10 min at 4 °C. An aliquot of the supernatant was retained as the input sample, while the remaining lysate was incubated overnight with target protein antibodies at concentrations recommended by the manufacturer. Protein A/G magnetic beads (Thermo Fisher, Waltham, MA, USA,88802) were then added and incubated at 4 °C for 4 h. The beads were washed three times with lysis buffer and subjected to western blot analysis, as described previously. All antibodies were listed in Supplementary Table 4.

### Generation of truncated mutations of CLPTM1L and ERLIN2

Flag-tagged CLPTM1L and HA-tag ERLIN2 were cloned into the pCDH plasmids. A series of truncated CLPTM1L or ERLIN2 mutants were generated based on wild-type CLPTM1L or ERLIN2, respectively: CLPTM1L-FLAG-ΔE1, lacking the first extracellular domain; CLPTM1L-FLAG-ΔC2/C3/C4, lacking the second to the fourth cytoplasmic domain; ERLIN2-FLAG-ΔL1-L5, lacking the L1 to L5 subregion of the lumenal domain. The primer sequences used for these constructs were provided in Supplementary Table 3.

### Immunohistochemistry (IHC)

Tissue sections were deparaffinized in xylene and rehydrated through a series of graded ethanol solutions (100%, 95%, 80%, and 70%). Antigen retrieval was conducted in EDTA solution using a microwave oven. After cooling to room temperature, the sections were rinsed with phosphate-buffered saline (PBS) and blocked with 5% goat serum for 1 h at room temperature. Primary antibodies, prepared according to the manufacturer’s instructions, were applied and incubated overnight at 4 °C. Following PBS washes, the sections were incubated with secondary antibodies for 30 min at room temperature. Chromogenic detection was performed using diaminobenzidine (DAB), followed by hematoxylin counterstaining. The sections were then mounted with neutral resin and visualized under a microscope (Olympus, Tokyo, Japan). All antibodies were listed in Supplementary Table 4.

### Luciferase reporter assays

The promoter region of CLPTM1L (2000 bp upstream of the transcription start site) was cloned into pGL3 basic plasmids. Cells were seeded in 24-well plates to reach approximately 70% confluence before transfection. Transfections were performed with KLF1 overexpression plasmids or KLF1-targeting siRNA, along with a Renilla luciferase plasmid as a control. 8 hours post-transfection, cells were further transfected with plasmids containing the CLPTM1L promoter. 48 hours after the final transfection, fluorescence was measured using the Dual-Luciferase Assay Kit (Promega, Madison, WI, USA, E1909) following the manufacturer’s instructions. Specific primers were listed in Supplementary Table 3.

### Chromatin Immunoprecipitation (ChIP)

S26 cells were transiently transfected with KLF1-HA plasmids. 48 hours post-transfection, the cells were harvested and resuspended in 1% formaldehyde for cross-linking on a shaker for 10 minutes. The cross-linking reaction was quenched with 125 mM glycine. The cell suspension was then centrifuged at 1000 rpm for 10 min at 4 °C, and the supernatant was discarded. Chromatin immunoprecipitation (ChIP) was performed using the SimpleChIP® Plus Enzymatic Chromatin IP Kit (Cell Signaling Technology, Danvers, USA, 9005), following the manufacturer’s protocol. The cleared lysates were incubated overnight at 4 °C with anti-HA antibody or IgG control (Cell Signaling Technology, Danvers, USA, 2729). qPCR was conducted using primers specific to the target genomic locus to analyze the bound DNA. The immunoprecipitation signals are expressed as a percentage of the total input chromatin. Percent input was calculated using the formula: Percent Input = 2% × 2^(C[T] 2%^ ^Input Sample – C[T] IP Sample)^, where C[T] represents the threshold cycle of the PCR reaction. Specific primers were listed in Supplementary Table 3.

### Cell proliferation assays

For growth curve analysis, cell suspensions were prepared at a density of 1,500 cells per 100 μL, with 100 μL seeded per well in 96-well plates. At 10 h post-seeding, CCK-8 reagent (Dojindo, Kumamoto, Japan, CK04) was added to the first group of wells, and optical density (OD) was measured at 450 nm using a microplate reader (Tecan, Männedorf, Switzerland). OD measurements were taken daily for three consecutive days. For colony formation assays, 1000 cells were seeded per well in 6-well plates and incubated for one week to allow for colony formation. Colonies were fixed with 4% paraformaldehyde and stained with crystal violet, and images were captured using a Bio-Rad ChemiDoc Touch Imaging System (Hercules, CA, USA). For EdU assays, cell proliferation was assessed using the BeyoClick™ EdU Cell Proliferation Kit with Alexa Fluor 488 (Beyotime, Shanghai, China, C0071), following the manufacturer’s protocol to evaluate fluorescence efficiency.

### Transwell migration and invasion assay

Transwell chambers (Corning, NY, USA, 3422) were inserted into wells containing 500 μL of complete medium. A suspension of 1 × 10^5^ cells in 200 μL of serum-free medium was added to the upper chamber coated with (invasion) or without (migration) Matrigel. After 20 h of incubation at 37 °C with 5% CO_2_, cells that had migrated to the lower surface of the membrane were fixed with 4% paraformaldehyde and stained with crystal violet. The number of migrated cells was then quantified using microscopy.

### Wound healing assay

A suspension of 1 × 10^5^ cells in 70 μL of culture medium was added to the scratcher chamber, which was affixed on the bottom of the culture dish. Following cell attachment to the surface, the scratcher was removed, and serum-free medium was added. The initial cell spacing was captured and recorded using a microscope. After 20 h of incubation at 37 °C with 5% CO_2_, the cell gap was measured again, and the change in gap distance was calculated.

### Immunofluorescence

Cells cultured in glass-bottom dishes were fixed with 4% paraformaldehyde and permeabilized using 0.1% Triton X-100. Blocking was performed with 5% sheep serum in 0.1% Triton X-100 for 1 hour at room temperature. The cells were then incubated with primary antibodies overnight at 4 °C, followed by incubation with fluorescent dye-conjugated secondary antibodies for 1 h at room temperature. Fluorescent images were acquired using a Carl Zeiss LSM 880 confocal microscope (Jena, Germany).

### RNA-seq analysis

Total RNA was extracted from cells using the RNeasy Mini Kit (Qiagen, Duesseldorf, Germany) according to the manufacturer’s protocol. RNA quantity was measured with the Qubit® 2.0 Fluorometer (Invitrogen, California, USA). Library preparation was performed using the TruSeq RNA Library Prep Kit (Illumina, San Diego, USA), following the manufacturer’s instructions. High-throughput sequencing of 150 base pair paired-end reads was conducted on the HiSeq X sequencer (Illumina, San Diego, USA). The high-quality paired-end reads were mapped to the reference genome using Bowtie 2 [35]. After ribosomal RNA sequences were removed, gene-specific read counts were quantified using HTseq [36]. Gene expression levels were normalized to transcripts per million (TPM), and genes with low expression (mean TPM < 2) across all datasets were excluded from further analyses.

### Free fatty acid assay

A total of 1 × 10^6^ cells were collected and lysed in 1% Triton X-100 in chloroform. The organic phase was separated by centrifugation at 13,000 × *g* for 10 min and subsequently dried using a vacuum centrifuge. Free fatty acid quantification was conducted using the Free Fatty Acid Quantitation Kit (Sigma-Aldrich, St. Louis, USA, MAK044) following the manufacturer’s protocol.

### Statistical analyses

All statistical data are presented as means ± S.D. Statistical data analysis was performed using GraphPad Prism v.6.01 (GraphPad Software). Significance was calculated by one-way ANOVA or Two-tailed unpaired Student’s t test (*P < 0.05, **P < 0.01, ***P < 0.001, ****P < 0.0001). Survival curves were calculated by Kaplan–Meier methods, with comparisons using the log-rank test.

## Supplementary information

Supplementary Figures

Supplementary Tables

Original Western Data

## Data Availability

The uncropped western blot data was provided in the Supplementary Material. Essential data was deposited in the Research Data Deposit public platform (RDD, RDDB2025156949; http://www.researchdata.org.cn).
