# Correction: CLPTM1L interacts with ERLIN2 to stabilize SREBP1 and drive tumorigenesis in nasopharyngeal carcinoma

**DOI:** 10.1038/s41419-025-07955-9

**Published:** 2025-08-26

**Authors:** Jian Zhang, Shu-Qiang Liu, Xiang-Yu Xiong, Ya-Qing Zhou, Pan-Pan Wei, Xin-Yuan Guan, Jin-Xin Bei, Qian Cui, Chun-Ling Luo

**Affiliations:** 1 The Eighth Affiliated Hospital, Sun Yat-sen University, Shenzhen, China; 2 State Key Laboratory of Oncology in South China, Guangdong Key Laboratory of Nasopharyngeal Carcinoma Diagnosis and Therapy, Guangdong Provincial Clinical Research Center for Cancer, Collaborative Innovation Center for Cancer Medicine, Sun Yat-sen University Cancer Center, Guangzhou, China; 3 Department of Clinical Oncology, The University of Hong Kong, Hong Kong, China; 4 Department of Medical Oncology, National Cancer Centre Singapore, Singapore, Singapore; 5 Department of Pathology, Guangdong Provincial People’s Hospital (Guangdong Academy of Medical Sciences), Southern Medical University, Guangzhou, Guangdong China

**Keywords:** Oncogenes, Head and neck cancer

Correction to: *Cell Death and Disease* 10.1038/s41419-025-07635-8, published online 23 June 2025

In this article the author order was given erroneously as:

Jian Zhang^1,2^, Shu-Qiang Liu^2^, Xiang-Yu Xiong^2^, Ya-Qing Zhou^2^, Pan-Pan Wei^2^, Xin-Yuan Guan^3^, Jin-Xin Bei^2,4^, Chun-Ling Luo^2,*^ and Qian Cui^5,**^

Initially it was given as:

Jian Zhang, Shu-Qiang Liu, Xiang-Yu Xiong, Ya-Qing Zhou, Pan-Pan Wei, Xin-Yuan Guan, Jin-Xin Bei, Qian Cui*, Chun-Ling Luo*

The original article has been corrected.

