# Supplementary material for: CLPTM1L interacts with ERLIN2 to stabilize SREBP1 and drive tumorigenesis in nasopharyngeal carcinoma
Source: Cell Death Dis. 2025 Jun 23;16(1):464. doi: 10.1038/s41419-025-07635-8 (PMC12185749; doi:10.1038/s41419-025-07635-8)
Supplement: Supplementary file 1 — Supplementary Figures [file 41419_2025_7635_MOESM1_ESM.docx]

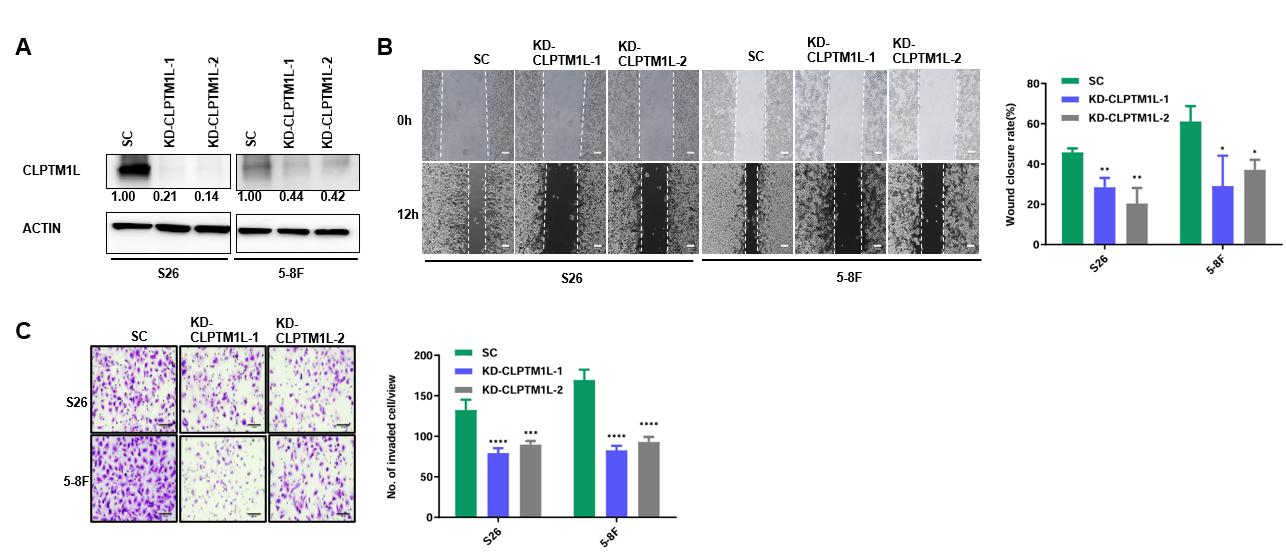


**Figure S1.** **Knockdown of CLPTM1L inhibits migration and invasion capabilities of NPC cells. A:** Western blot analysis demonstrating the expression of CLPTM1L in S26 and 5-8F cells transfected with CLPTM1L siRNAs (KD-CLPTM1L-1/2) or control siRNA (SC). ACTIN is used as an internal control. **B-C:** Wound healing assay (B) and Transwell assay (C) showing the migration and invasion abilities of cells described in (A). Statistical analysis is performed by one-way ANOVA and data are presented as the mean± SD. Scale bars, 100 μm. *, *P* < 0.05; **, *P* < 0.01; ***, *P* < 0.001; ****, *P*˂0.0001.


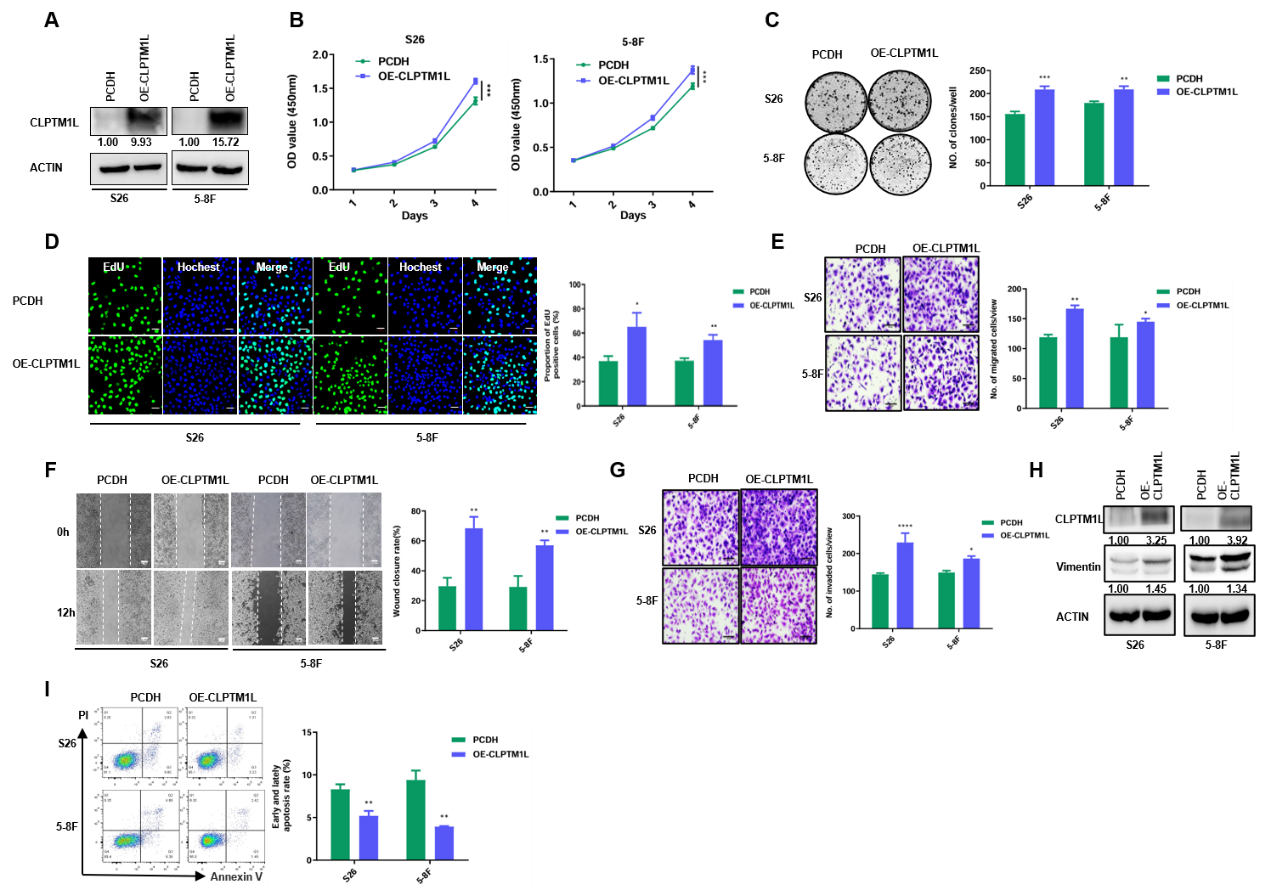


**Figure S2. CLPTM1L drives oncogenic phenotypes in NPC. A:** Western blot analysis demonstrating the expression of CLPTM1L in S26 and 5-8F cells infected with lentivirus expressing CLPTM1L or overexpression vectors (PCDH). ACTIN is used as an internal control. **B-C:** CCK8 assay (B) and colony formation assay (C) measuring the proliferation abilities of cells described in (A). **D:** Representative images of EdU staining assay with cells described in (A), with statistical analysis presented at the right. **E-F:** Transwell assay (E) and wound healing assay (F) showing the migration abilities of cells described in (A). **G:** Transwell assay showing the invasion abilities of cells described in (A). **H:** Western blotting analysis demonstrating the protein levels of CLPTM1L and Vimentin of CLPTM1L-overexpressing cells described in (A). ACTIN is used as an internal control. **F:** Flow cytometry analysis with FITC Annexin V and PI in cells described in (A). Statistical analysis is performed by Student’s t-test and data are presented as the mean± SD. Scale bars, 100 μm. *, *P* < 0.05; **, *P* < 0.01; ***, *P* < 0.001; ****, *P*˂0.0001.


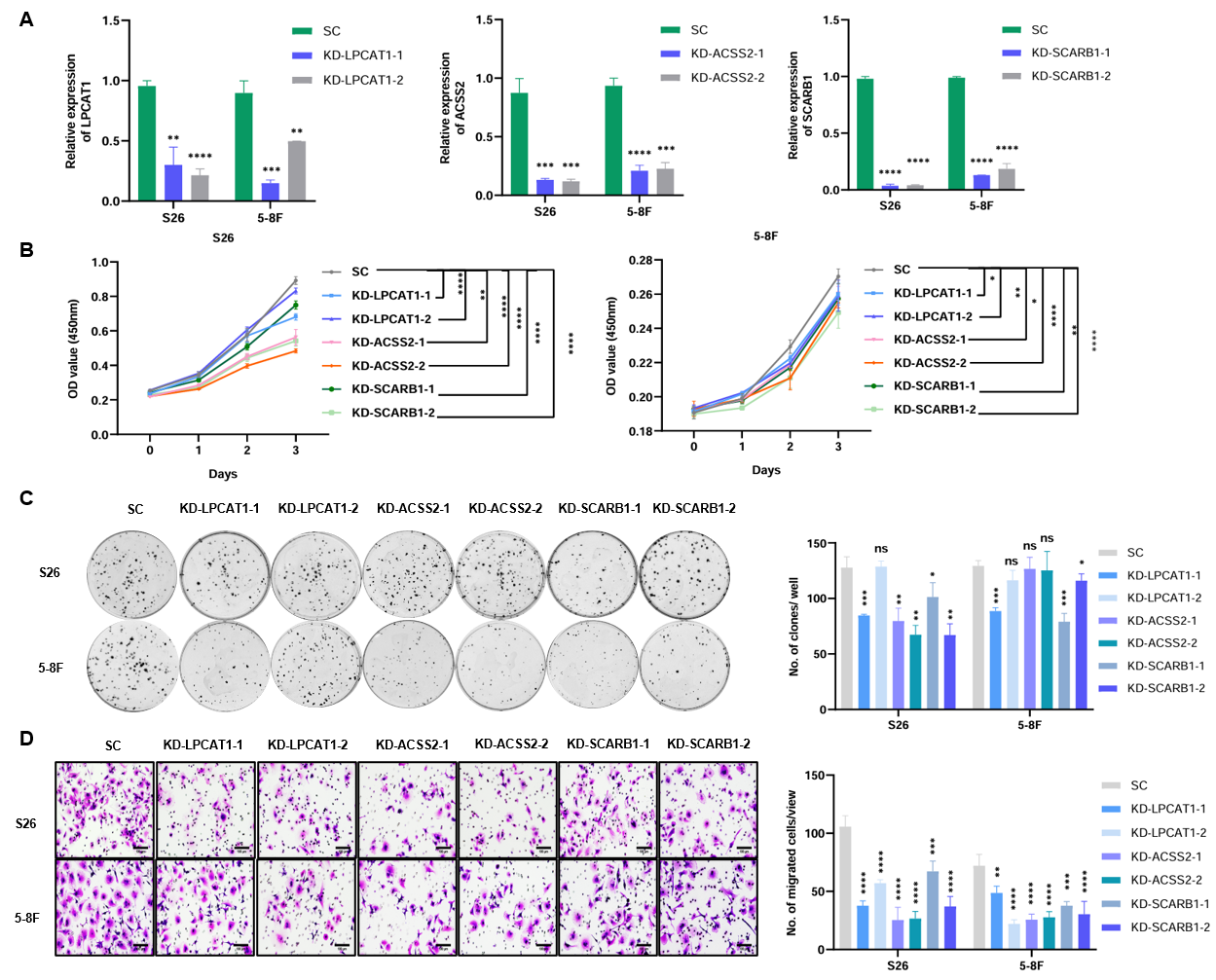


**Figure S3. Knockdown of LPCAT1, ACSS2, or SCARB1 inhibits the proliferation and migration capacities of NPC cells.** **A:** RT-qPCR analyses showing the knockdown efficiency of either LPCAT1, ACSS2 or SCARB1 in NPC cells transfected with indicated siRNAs (KD-1/2) or control siRNA (SC). **B:** CCK8 assay measuring the proliferation abilities of cells described in (A). **C:** Colony formation assay with cells described in (A), with statistics presented at the right. **D:** Transwell assay showing the migration abilities of cells described in (A), alongside with statistics at the right. Differences between groups are analyzed by one-way ANOVA and data are presented as the mean± SD. *, *P* < 0.05; **, *P* < 0.01; ***, *P* < 0.001; ****, *P*˂0.0001. Scale bars, 100 μm.


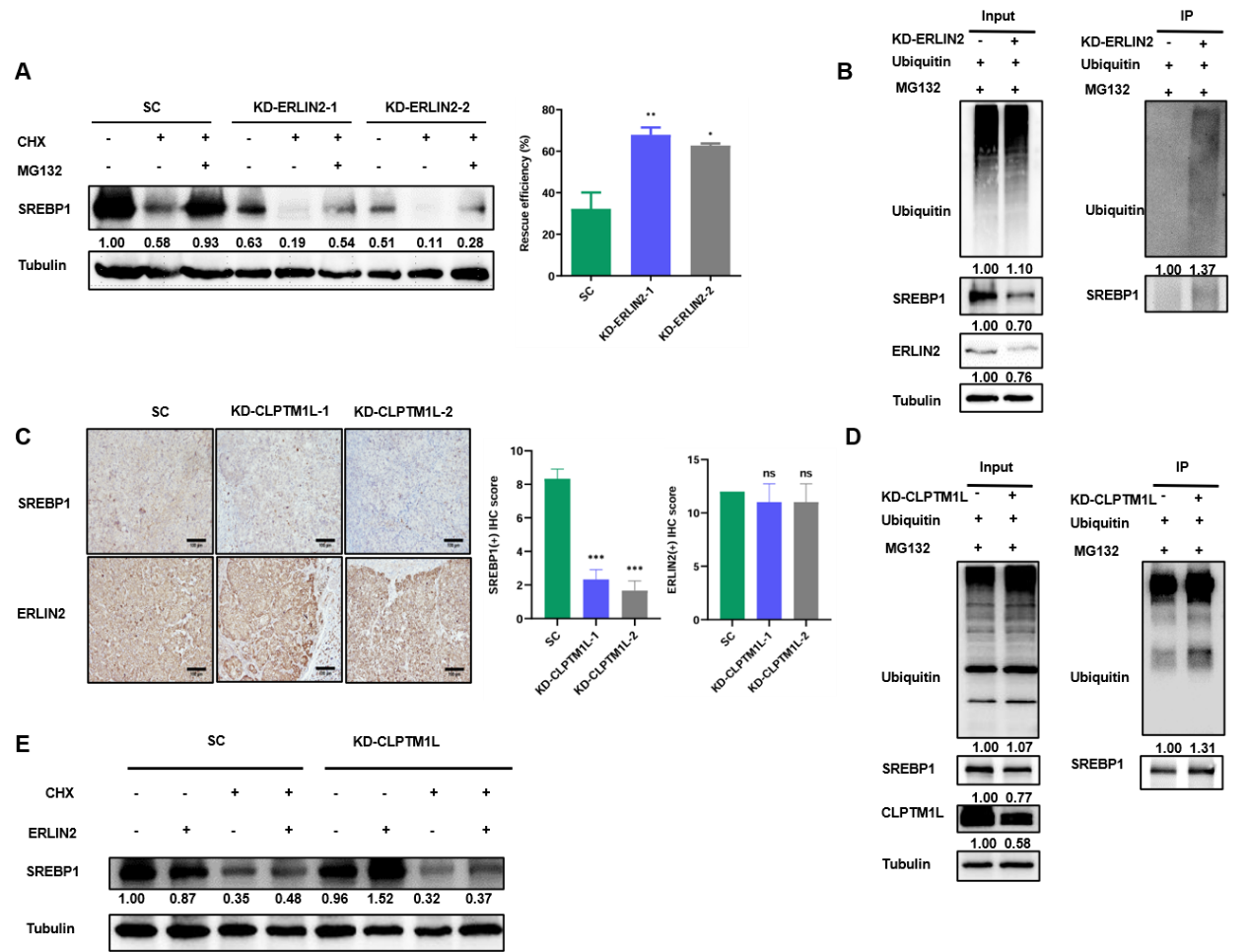


**Figure S4. CLPTM1L and ERLIN2 jointly stabilize SREBP1 protein by inhibiting its ubiquitination. A:** S26 cells are transfected with siRNAs targeting ERLIN2 or control siRNA, followed by treatment with cycloheximide (CHX) and MG132. Western blot analysis (left) shows the levels of SREBP1 protein, and the bar graph (right) quantifies the rescue efficiency of SREBP1 after MG132 treatment. **B:** Immunoprecipitation with anti-SREBP1 antibodies in S26 cells transfected with ERLIN2 siRNAs, alongside HA-ubiquitin. All the samples are treated with MG132. **C:** Representative images of IHC staining showing the protein levels of SREBP1 and ERLIN2 in subcutaneous tumors derived from CLPTM1L-knockdown NPC cells or control cells, and the statistical analysis of IHC intensity as scoring is presented on the right. **D:** Immunoprecipitation with anti-SREBP1 antibodies in S26 cells transfected with CLPTM1L siRNAs, alongside HA-ubiquitin. All the samples are treated with MG132. **E:** Western blot analysis of SREBP1 levels in S26 cells simultaneously knock downing CLPTM1L and overexpressing ERLIN2, and subsequently treated with or without CHX for 4 h. The “+” and “-” indications represent the presence or absence, respectively. Statistical analysis is performed by one-way ANOVA and data are presented as the mean± SD. Scale bars, 100 μm. *, *P* < 0.05; **, *P* < 0.01; ***, *P* < 0.001.


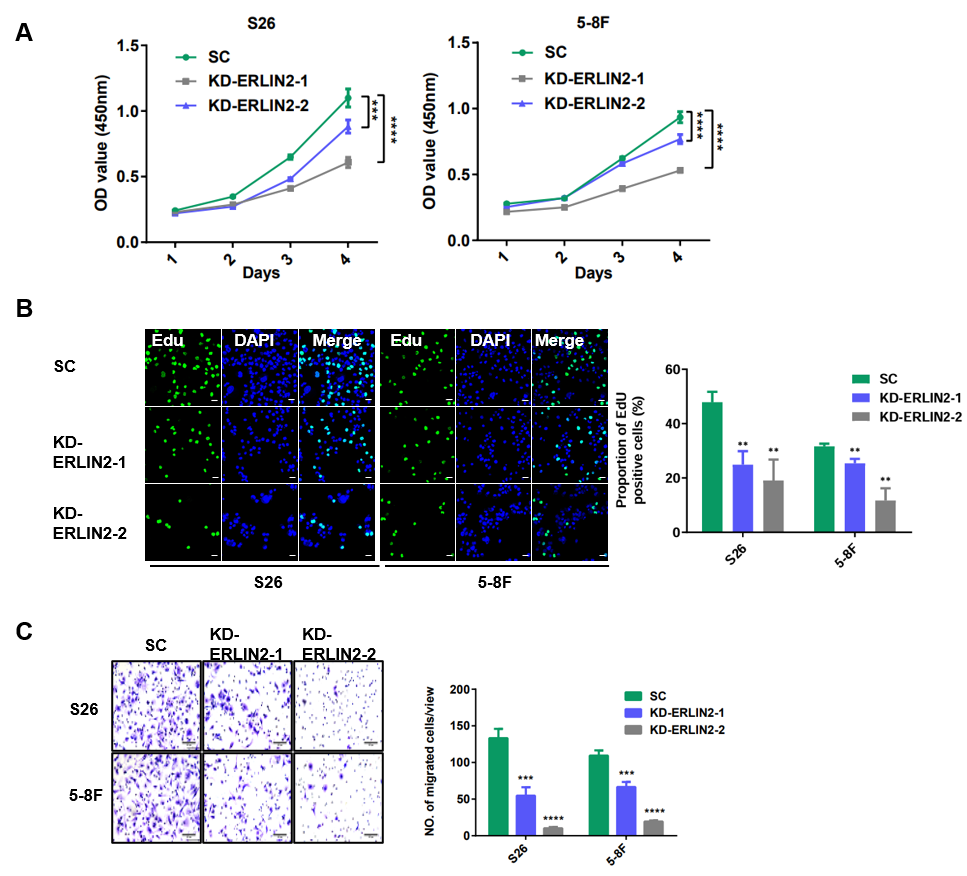


**Figure S5. ERLIN2 knockdown inhibits the proliferation and migration capacities in NPC cells. A-B:** CCK8 (A) and EdU staining assay (B) showing the proliferation abilities of S26 and 5-8F cells transfected with ERLIN2 siRNAs or control siRNA. **C:** Transwell assay demonstrating the migration capacities of cells described in (A). Statistical analysis is performed by one-way ANOVA and data are presented as the mean ± SD. Scale bars, 100 μm. **, *P* < 0.01; ***, *P* < 0.001; ****, *P*˂0.0001.


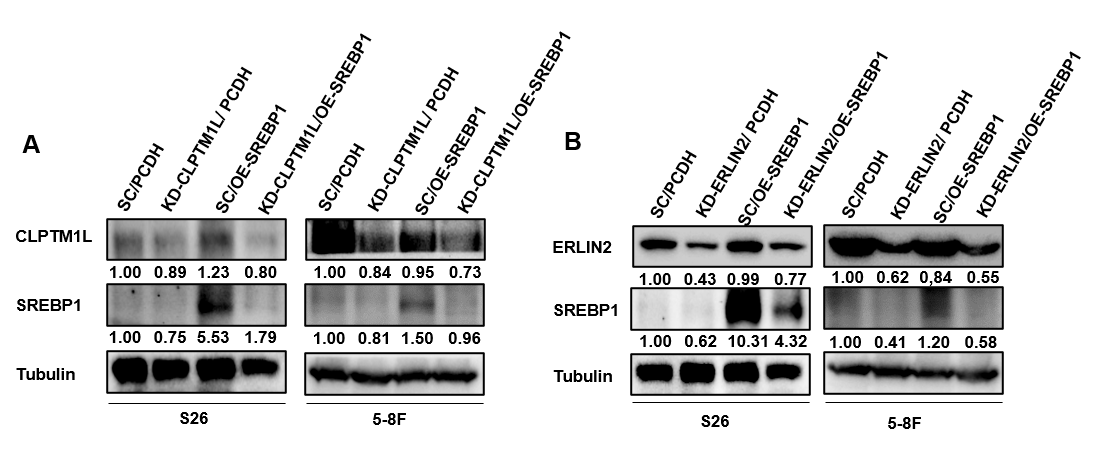


**Figure S6. SREBP1 overexpression restores the downregulated protein levels caused by knockdowns of CLPTM1L or ERLIN2. A:** Western blot analysis demonstrating the protein levels of CLPTM1L and SREBP1 in S26 and 5-8F cells stably expressing CLPTM1L shRNAs or control shRNA, followed by infection with lentivirus expressing SREBP1. Tubulin is used as an internal control. **B:** Western blot analysis showing the protein levels of ERLIN2 and SREBP1 in S26 and 5-8F cells stably expressing ERLIN2 shRNAs or control shRNA, followed by infection with lentivirus expressing SREBP1. Tubulin is used as an internal control.


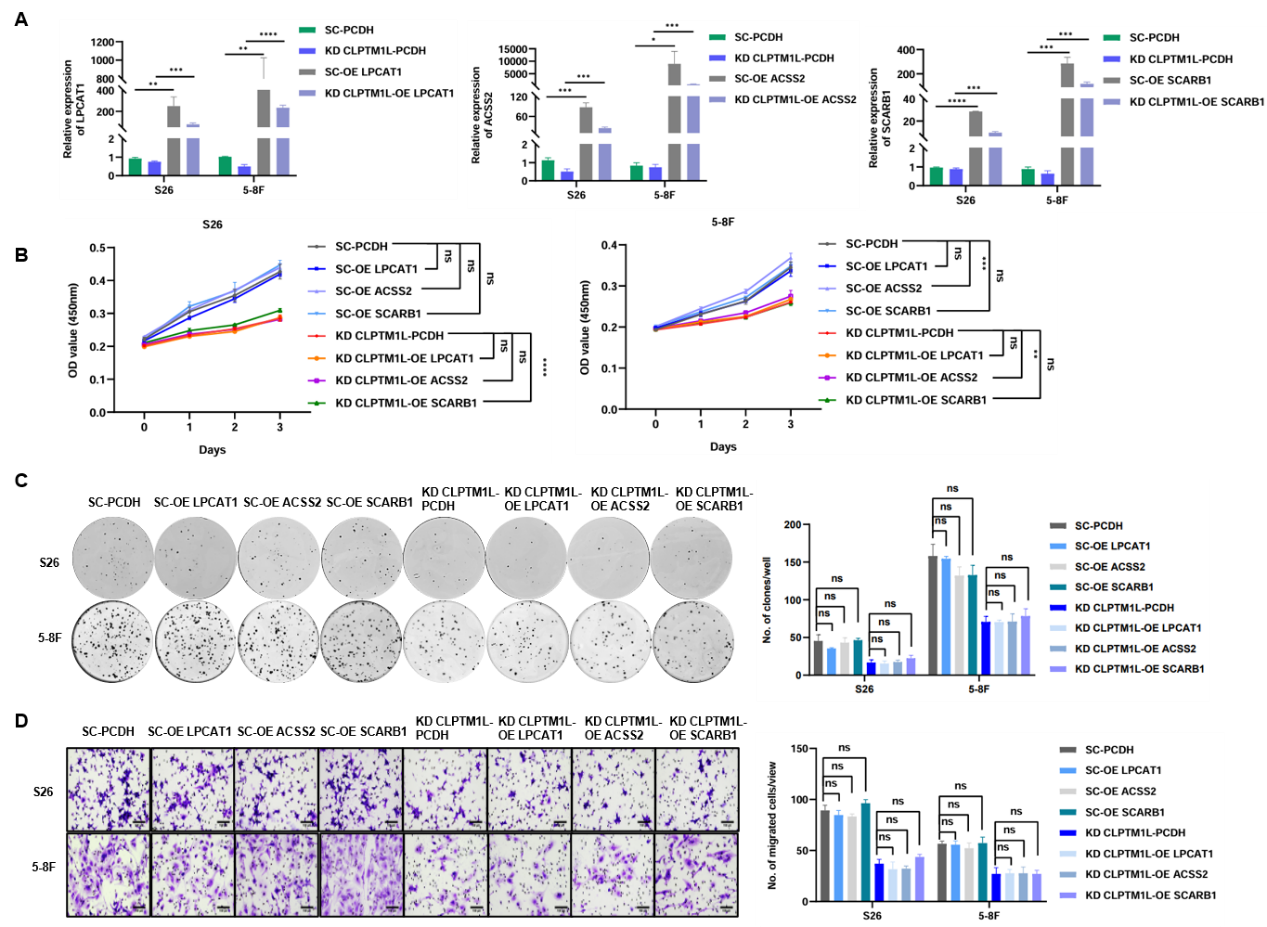


**Figure S7. LPCAT1, ACSS2, or SCARB1 fails to rescue phenotypes caused by CLPTM1L knockdown. A:** RT-qPCR analyses showing the relative mRNA expression of in S26 and 5-8F cells stably expressing CLPTM1L shRNAs or control shRNA, and simultaneously expressed with either LPCAT1, ACSS2, SCARB1 or control (PCDH). **B:** CCK8 assay measuring the proliferation abilities of cells described in (A). **C:** Colony formation assay with cells described in (A), with statistics presented at the right. **D:** Transwell assay showing the migration abilities of cells described in (A), alongside with statistics at the right. Differences between groups are analyzed by one-way ANOVA and data are presented as the mean± SD. *, *P* < 0.05; **, *P* < 0.01; ***, *P* < 0.001; ****, *P*˂0.0001. Scale bars, 100 μm.
