# Supplementary material for: CLPTM1L interacts with ERLIN2 to stabilize SREBP1 and drive tumorigenesis in nasopharyngeal carcinoma
Source: Cell Death Dis. 2025 Jun 23;16(1):464. doi: 10.1038/s41419-025-07635-8 (PMC12185749; doi:10.1038/s41419-025-07635-8)
Supplement: Supplementary file 2 — Supplementary Tables [file 41419_2025_7635_MOESM2_ESM.docx]

**Supplementary Table 1. Clinical characteristics of NPC patients (n=107).**

| **ID** | **Overall survival time (month)** | **CLPTM1L IHC score** |
| --- | --- | --- |
| 1 | 80 | 0.05 |
| 2 | 73 | 0.05 |
| 3 | 85 | 0.1 |
| 4 | 70 | 0.1 |
| 5 | 68 | 0.15 |
| 6 | 85 | 0.2 |
| 7 | 71 | 0.2 |
| 8 | 85 | 0.3 |
| 9 | 78 | 0.3 |
| 10 | 81 | 0.3 |
| 11 | 71 | 0.3 |
| 12 | 69 | 0.3 |
| 13 | 67 | 0.3 |
| 14 | 59 | 0.3 |
| 15 | 66 | 0.3 |
| 16 | 79 | 0.4 |
| 17 | 81 | 0.4 |
| 18 | 70 | 0.4 |
| 19 | 66 | 0.4 |
| 20 | 73 | 0.45 |
| 21 | 82 | 0.55 |
| 22 | 34 | 0.6 |
| 23 | 84 | 0.65 |
| 24 | 85 | 0.8 |
| 25 | 84 | 0.8 |
| 26 | 84 | 0.8 |
| 27 | 82 | 0.8 |
| 28 | 65 | 0.8 |
| 29 | 72 | 0.8 |
| 30 | 72 | 0.8 |
| 31 | 66 | 0.8 |
| 32 | 85 | 0.85 |
| 33 | 81 | 0.85 |
| 34 | 66 | 0.85 |
| 35 | 84 | 0.9 |
| 36 | 83 | 0.9 |
| 37 | 75 | 0.9 |
| 38 | 81 | 0.9 |
| 39 | 81 | 0.9 |
| 40 | 80 | 0.9 |
| 41 | 79 | 0.9 |
| 42 | 74 | 0.9 |
| 43 | 74 | 0.9 |
| 44 | 73 | 0.9 |
| 45 | 72 | 0.9 |
| 46 | 72 | 0.9 |
| 47 | 71 | 0.9 |
| 48 | 71 | 0.9 |
| 49 | 70 | 0.9 |
| 50 | 70 | 0.9 |
| 51 | 69 | 0.9 |
| 52 | 69 | 0.9 |
| 53 | 69 | 0.9 |
| 54 | 68 | 0.9 |
| 55 | 67 | 0.9 |
| 56 | 67 | 0.9 |
| 57 | 66 | 0.9 |
| 58 | 66 | 0.9 |
| 59 | 65 | 0.9 |
| 60 | 84 | 0.95 |
| 61 | 83 | 0.95 |
| 62 | 81 | 0.95 |
| 63 | 81 | 0.95 |
| 64 | 69 | 0.95 |
| 65 | 68 | 0.95 |
| 66 | 86 | 0.975 |
| 67 | 85 | 0.975 |
| 68 | 84 | 0.975 |
| 69 | 55 | 1.2 |
| 70 | 74 | 1.2 |
| 71 | 60 | 1.275 |
| 72 | 67 | 1.275 |
| 73 | 63 | 1.275 |
| 74 | 86 | 1.35 |
| 75 | 50 | 1.35 |
| 76 | 76 | 1.35 |
| 77 | 83 | 1.35 |
| 78 | 49 | 1.35 |
| 79 | 81 | 1.35 |
| 80 | 81 | 1.35 |
| 81 | 74 | 1.35 |
| 82 | 70 | 1.35 |
| 83 | 69 | 1.35 |
| 84 | 68 | 1.35 |
| 85 | 68 | 1.35 |
| 86 | 66 | 1.35 |
| 87 | 65 | 1.35 |
| 88 | 80 | 1.425 |
| 89 | 80 | 1.425 |
| 90 | 73 | 1.425 |
| 91 | 72 | 1.425 |
| 92 | 72 | 1.425 |
| 93 | 70 | 1.425 |
| 94 | 69 | 1.425 |
| 95 | 69 | 1.425 |
| 96 | 69 | 1.425 |
| 97 | 69 | 1.425 |
| 98 | 67 | 1.425 |
| 99 | 66 | 1.425 |
| 100 | 70 | 1.6 |
| 101 | 66 | 1.8 |
| 102 | 63 | 1.8 |
| 103 | 74 | 1.9 |
| 104 | 73 | 1.9 |
| 105 | 69 | 1.9 |
| 106 | 69 | 1.9 |
| 107 | 68 | 1.9 |

**Supplementary Table 2. List of CLPTM1L interacted proteins identified by IP-MS.**

| **Protein ID** | **Description** |
| --- | --- |
| 1 | Caveolae-associated protein 3 OS=Homo sapiens OX=9606 GN=CAVIN3 PE=1 SV=3 |
| 2 | Guanine nucleotide-binding protein G(s) subunit alpha isoforms short OS=Homo sapiens OX=9606 GN=GNAS PE=1 SV=1 |
| 3 | Transmembrane protein 43 OS=Homo sapiens OX=9606 GN=TMEM43 PE=1 SV=1 |
| 4 | Tropomodulin-3 OS=Homo sapiens OX=9606 GN=TMOD3 PE=1 SV=1 |
| 5 | Actin-related protein 2 OS=Homo sapiens OX=9606 GN=ACTR2 PE=1 SV=1 |
| 6 | Erlin-2 OS=Homo sapiens OX=9606 GN=ERLIN2 PE=1 SV=1 |
| 7 | 40S ribosomal protein SA OS=Homo sapiens OX=9606 GN=RPSA PE=1 SV=4 |
| 8 | Ubiquitin-60S ribosomal protein L40 OS=Homo sapiens OX=9606 GN=UBA52 PE=1 SV=2 |
| 9 | HLA class I histocompatibility antigen A alpha chain OS=Homo sapiens OX=9606 GN=HLA-A PE=1 SV=2 |
| 10 | Guanine nucleotide-binding protein subunit alpha-11 OS=Homo sapiens OX=9606 GN=GNA11 PE=1 SV=2 |
| 11 | Immunoglobulin gamma-1 heavy chain OS=Homo sapiens OX=9606 PE=1 SV=2 |
| 12 | Phosphoglycerate kinase 1 OS=Homo sapiens OX=9606 GN=PGK1 PE=1 SV=3 |
| 13 | TGF-beta receptor type-2 OS=Homo sapiens OX=9606 GN=TGFBR2 PE=1 SV=2 |
| 14 | HLA class I histocompatibility antigen B alpha chain OS=Homo sapiens OX=9606 GN=HLA-B PE=1 SV=3 |
| 15 | Casein kinase II subunit alpha 3 OS=Homo sapiens OX=9606 GN=CSNK2A3 PE=1 SV=2 |
| 16 | Putative heat shock 70 kDa protein 7 OS=Homo sapiens OX=9606 GN=HSPA7 PE=5 SV=2 |
| 17 | Myosin-9 OS=Homo sapiens OX=9606 GN=MYH9 PE=1 SV=4 |
| 18 | Serpin B6 OS=Homo sapiens OX=9606 GN=SERPINB6 PE=1 SV=3 |
| 19 | 7-dehydrocholesterol reductase OS=Homo sapiens OX=9606 GN=DHCR7 PE=1 SV=1 |
| 20 | Kunitz-type protease inhibitor 2 OS=Homo sapiens OX=9606 GN=SPINT2 PE=1 SV=2 |
| 21 | Integral membrane protein 2B OS=Homo sapiens OX=9606 GN=ITM2B PE=1 SV=1 |
| 22 | Stomatin-like protein 2 mitochondrial OS=Homo sapiens OX=9606 GN=STOML2 PE=1 SV=1 |
| 23 | Heterogeneous nuclear ribonucleoproteins C1/C2 OS=Homo sapiens OX=9606 GN=HNRNPC PE=1 SV=4 |
| 24 | POM121-like protein 12 OS=Homo sapiens OX=9606 GN=POM121L12 PE=2 SV=3 |
| 25 | Isocitrate dehydrogenase [NAD] subunit beta mitochondrial OS=Homo sapiens OX=9606 GN=IDH3B PE=1 SV=2 |
| 26 | Interferon gamma receptor 1 OS=Homo sapiens OX=9606 GN=IFNGR1 PE=1 SV=1 |
| 27 | Tetraspanin-3 OS=Homo sapiens OX=9606 GN=TSPAN3 PE=2 SV=1 |
| 28 | Poly(rC)-binding protein 3 OS=Homo sapiens OX=9606 GN=PCBP3 PE=1 SV=2 |
| 29 | Mitochondrial-processing peptidase subunit beta OS=Homo sapiens OX=9606 GN=PMPCB PE=1 SV=2 |
| 30 | Sequestosome-1 OS=Homo sapiens OX=9606 GN=SQSTM1 PE=1 SV=1 |
| 31 | Prospero homeobox protein 1 OS=Homo sapiens OX=9606 GN=PROX1 PE=1 SV=2 |
| 32 | Alpha-amylase 1C OS=Homo sapiens OX=9606 GN=AMY1C PE=1 SV=1 |
| 33 | Cytospin-B OS=Homo sapiens OX=9606 GN=SPECC1 PE=1 SV=1 |
| 34 | tRNA (guanine-N(7)-)-methyltransferase OS=Homo sapiens OX=9606 GN=METTL1 PE=1 SV=1 |
| 35 | Ecto-ADP-ribosyl transferase 4 OS=Homo sapiens OX=9606 GN=ART4 PE=2 SV=2 |
| 36 | Magnesium transporter MRS2 homolog mitochondrial OS=Homo sapiens OX=9606 GN=MRS2 PE=1 SV=1 |
| 37 | Elongator complex protein 5 OS=Homo sapiens OX=9606 GN=ELP5 PE=1 SV=2 |
| 38 | Aldehyde oxidase OS=Homo sapiens OX=9606 GN=AOX1 PE=1 SV=2 |
| 39 | Immunoglobulin heavy variable 6-1 OS=Homo sapiens OX=9606 GN=IGHV6-1 PE=3 SV=1 |
| 40 | Splicing factor 3A subunit 2 OS=Homo sapiens OX=9606 GN=SF3A2 PE=1 SV=2 |
| 41 | Rho-related GTP-binding protein RhoV OS=Homo sapiens OX=9606 GN=RHOV PE=1 SV=1 |
| 42 | 26S proteasome non-ATPase regulatory subunit 13 OS=Homo sapiens OX=9606 GN=PSMD13 PE=1 SV=2 |
| 43 | Vacuolar protein sorting-associated protein 13D OS=Homo sapiens OX=9606 GN=VPS13D PE=1 SV=2 |

**Supplementary Table 3. List of primers.**

| **Primer-id** | **Sequence (5'-3')** |
| --- | --- |
| NC-siRNA | TTCTCCGAACGTGTCACGT |
| CLPTM1L-siRNA-1 | CAGCGAATCTGAGAGGAAA |
| CLPTM1L-siRNA-2 | GCTGTATGCCTACATCTTC |
| ERLIN2-siRNA-1 | GCCTCTCCGGTACTAACAT |
| ERLIN2-siRNA-2 | GCCCTCATCTTCAACAAGA |
| SREBP1-siRNA-1 | GCACTTTCGAAGACATGCT |
| SREBP1-siRNA-2 | CCACTCCATTGAAGATGTA |
| LPCAT1- siRNA-1 | GGGAACTCTGATCCAGTATAT |
| LPCAT1- siRNA-2 | GGAAAGTGGCCACAGATAATG |
| ACSS2- siRNA-1 | GCCCATTCCTTCGGTACAACT |
| ACSS2- siRNA-2 | GGATTCCAGCTGCAGTCTTCT |
| SCARB1- siRNA-1 | GCGGTGATGATGGAGAATAAG |
| SCARB1- siRNA-2 | GCACTGTGGGTGAGATCATGT |
| KLF1-siRNA-1 | CAGGATGACTTCCTCAAGT |
| KLF1-siRNA-2 | CTTCCTGAGTTGTTTGGGA |
| CLPTM1L-qPCR-F | GGAAAACCGTGCATTACCTGCC |
| CLPTM1L-qPCR-R | CAGTGAGACCTTGTCGTAGGAC |
| ERLIN2-qPCR-F | ACGCTTCAAGAGGTCTACATTG |
| ERLIN2-qPCR-R | ATTGCCTCTGGTATGTTGGG |
| SREBP1-qPCR-F | GCACTTTCGAAGACATGCT |
| SREBP1-qPCR-R | AGGTTCCAGAGGAGGCTACAAG |
| KLF1-qPCR-F | TTGCGGCAAGAGCTACACCAAG |
| KLF1-qPCR-R | GTAGTGGCGGGTCAGCTCGTC |
| LPCAT1-qPCR-F | CGACCTATTCCGAGCCATTGAC |
| LPCAT1-qPCR-R | GTGAGGTCTCTGCACAGCTTTC |
| ACSS2-qPCR-F | GGTGACCAAGTTCTACACAGCAC |
| ACSS2-qPCR-R | GTTCACCCACTGTGCCTAACAC |
| SCARB1-qPCR-F | GGTCCAGAACATCAGCAGGATC |
| SCARB1- qPCR-R | GCCACATTTGCCCAGAAGTTCC |
| ACTIN-qPCR-F | CCCACACTGTGCCCATCTAC |
| ACTIN-qPCR-R | GGAACCGCTCATTGCCAATG |
| CLPTM1L promoter-F | CTCTTTCCTCTTTTGTTTG |
| CLPTM1L promoter-R | TGCAGAAGCAGACGCAGT |
| IRF1-siRNA | GGCTAGAGATGCAGATTAA |
| TCF4-siRNA | CTGGATTTCAGTGCGATGT |
| NR2F1-siRNA | CCCAACAACATTATGGGCA |
| BHLHE40 -siRNA | CCCACATGTACCAAGTGTA |
| IRF1-siRNA | GGCTAGAGATGCAGATTAA |
| ZNF135-siRNA | CCCAGAGATTAAGGGACAT |
| KLF1-chipPCR-F | TGAGGGTTCTGAGCACTTCGTTT |
| KLF1-chipPCR-R | CCTGGTGGAAGGTGATACTGGAA |
| CLPTM1L-HA-de36-280-F | GCATCGTCTACACCCGCCCGGATACCAACTTATACTTCCT |
| CLPTM1L-HA-de36-280-R | AGGAAGTATAAGTTGGTATCCGGGCGGGTGTAGACGATGC |
| CLPTM1L-HA-de309-321-F | TCCTGGCCTTTAAAAATGACTCCACCAAGGCAGTGCTCTG |
| CLPTM1L-HA-de309-321-R | CAGAGCACTGCCTTGGTGGAGTCATTTTTAAAGGCCAGGA |
| CLPTM1L-HA-de368-399-F | TGTGGAAAGTGAAGAAGGCAGCCATGAAGTACTTGTCATA |
| CLPTM1L-HA-de368-399-R | TATGACAAGTACTTCATGGCTGCCTTCTTCACTTTCCACA |
| CLPTM1L-HA-de453-536-F | TCTTCATGCTGCCCCAGCTCACGGACTACCCATACGATGT |
| CLPTM1L-HA-de453-536-R | ACATCGTATGGGTAGTCCGTGAGCTGGGGCAGCATGAAGA |
| ERLIN2-Flag-de26-100-F | TCTTCTCAGCTGTGCACAAGTATGATATAGTGAAGAACTA |
| ERLIN2-Flag-de26-100-R | TAGTTCTTCACTATATCATACTTGTGCACAGCTGAGAAGA |
| ERLIN2-Flag-de101-176-F | TCCTGGTCCCGAACGCAGTGGAGGCAATCCGCAGAAACTA |
| ERLIN2-Flag-de101-176-R | TAGTTTCTGCGGATTGCCTCCACTGCGTTCGGGACCAGGA |
| ERLIN2-Flag-de177-230-F | TAACAAAGCCCAACATACCACAGAAGGTGATGGAGAAGGA |
| ERLIN2-Flag-de177-230-R | TCCTTCTCCATCACCTTCTGTGGTATGTTGGGCTTTGTTA |
| ERLIN2-Flag-de231-274-F | TGGCTGAGATCACCTACGGGAAGCTAACCCCTGAATATCT |
| ERLIN2-Flag-de231-274-R | AGATATTCAGGGGTTAGCTTCCCGTAGGTGATCTCAGCCA |
| ERLIN2-Flag-de275-339-F | TAGCCGAAGCCAATAAGCTGAAGGAGAATTGAGTCGAC |
| ERLIN2-Flag-de275-339-R | GTCGACTCAATTCTCCTTCAGCTTATTGGCTTCGGCTA |

**Supplementary Table 4. List of antibodies.**

| **Antibody** | **Company** | **Catalog Number** |
| --- | --- | --- |
| Anti-mouse IgG, HRP-linked antibody | Cell Signaling Technology | 7076 |
| Anti-rabbit IgG, HRP-linked Antibody | Cell Signaling Technology | 7074 |
| CLPTM1L | Sigma-Aldrich | HPA014791 |
| E-cadherin | Cell Signaling Technology | 14472 |
| Vimentin | Cell Signaling Technology | 5741 |
| PARP1 | Cell Signaling Technology | 9542 |
| Cleaved-PARP1 | Cell Signaling Technology | 5625 |
| ACTIN | Cell Signaling Technology | 3700 |
| Tublin | Cell Signaling Technology | 2148 |
| HA-tag | Sigma-Aldrich | H6908 |
| Flag-tag | Sigma-Aldrich | F1804 |
| ERLIN2 | Abcam | ab128924 |
| SREBP1 | Santa Cruz Biotechnology | sc-13551 |
| Goat anti-Rabbit IgG (H+L) Cross-Adsorbed Secondary Antibody, Alexa Fluor™ 488 | Invitrogen | A11004 |
| Goat anti-Mouse IgG (H+L) Cross-Adsorbed Secondary Antibody, Alexa Fluor™ 568 | Invitrogen | A11008 |
| GAPDH | Abclonal | AC002 |
| Ubiquitin | Cell Signaling Technology | 58395 |
