# Supplementary figures and images for: CLPTM1L interacts with ERLIN2 to stabilize SREBP1 and drive tumorigenesis in nasopharyngeal carcinoma
Source: Cell Death Dis. 2025 Jun 23;16(1):464. doi: 10.1038/s41419-025-07635-8 (PMC12185749; doi:10.1038/s41419-025-07635-8)

## Figure 1

# B

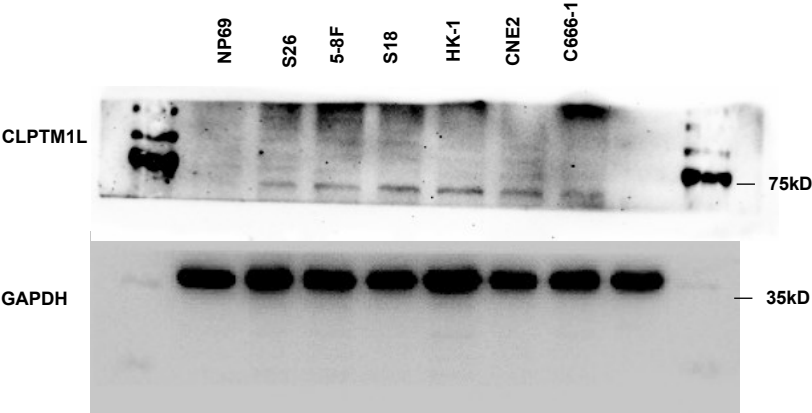

Figure 2

E

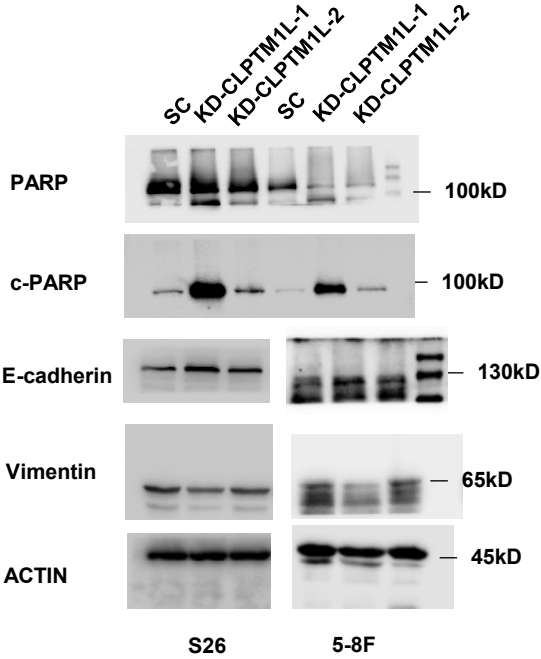

Figure 5

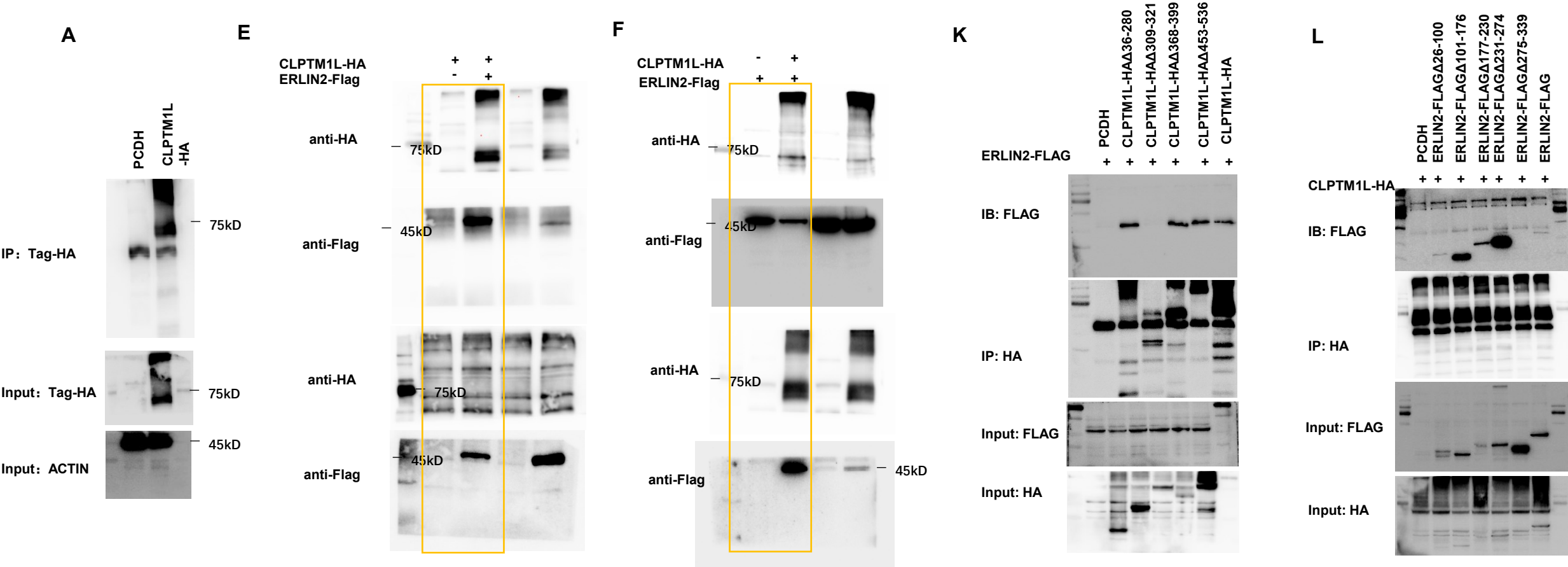

Figure 6

B

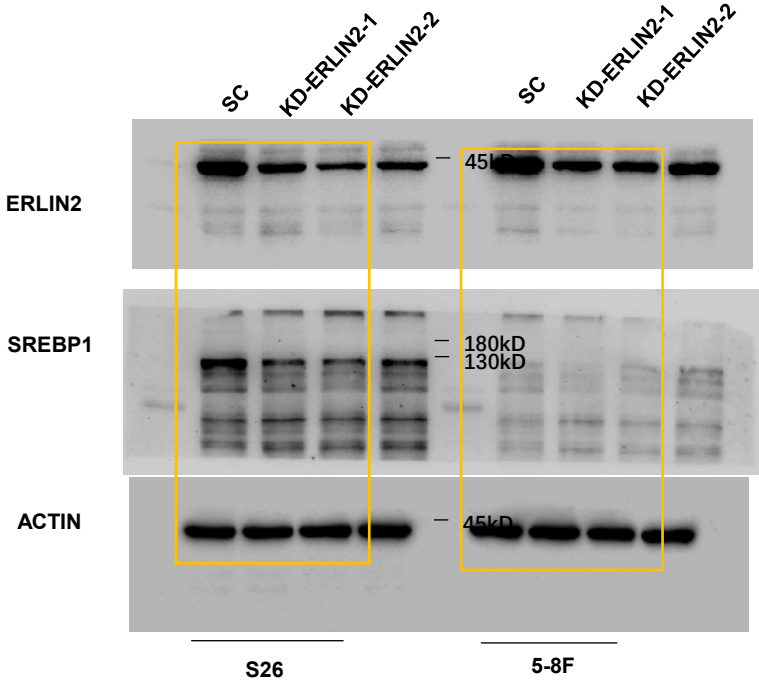

C

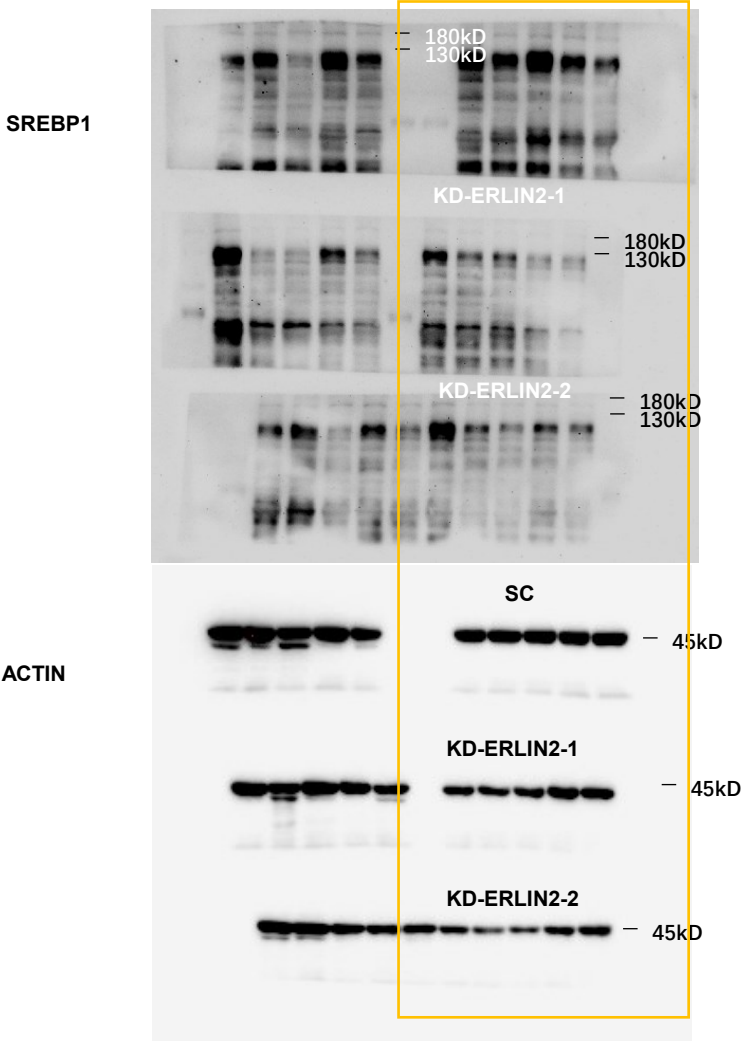

D

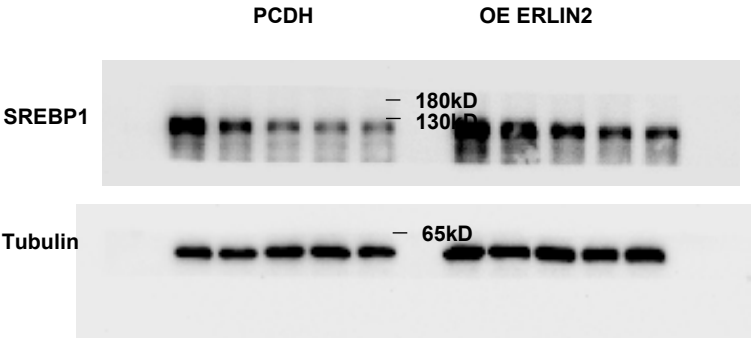

Figure 6

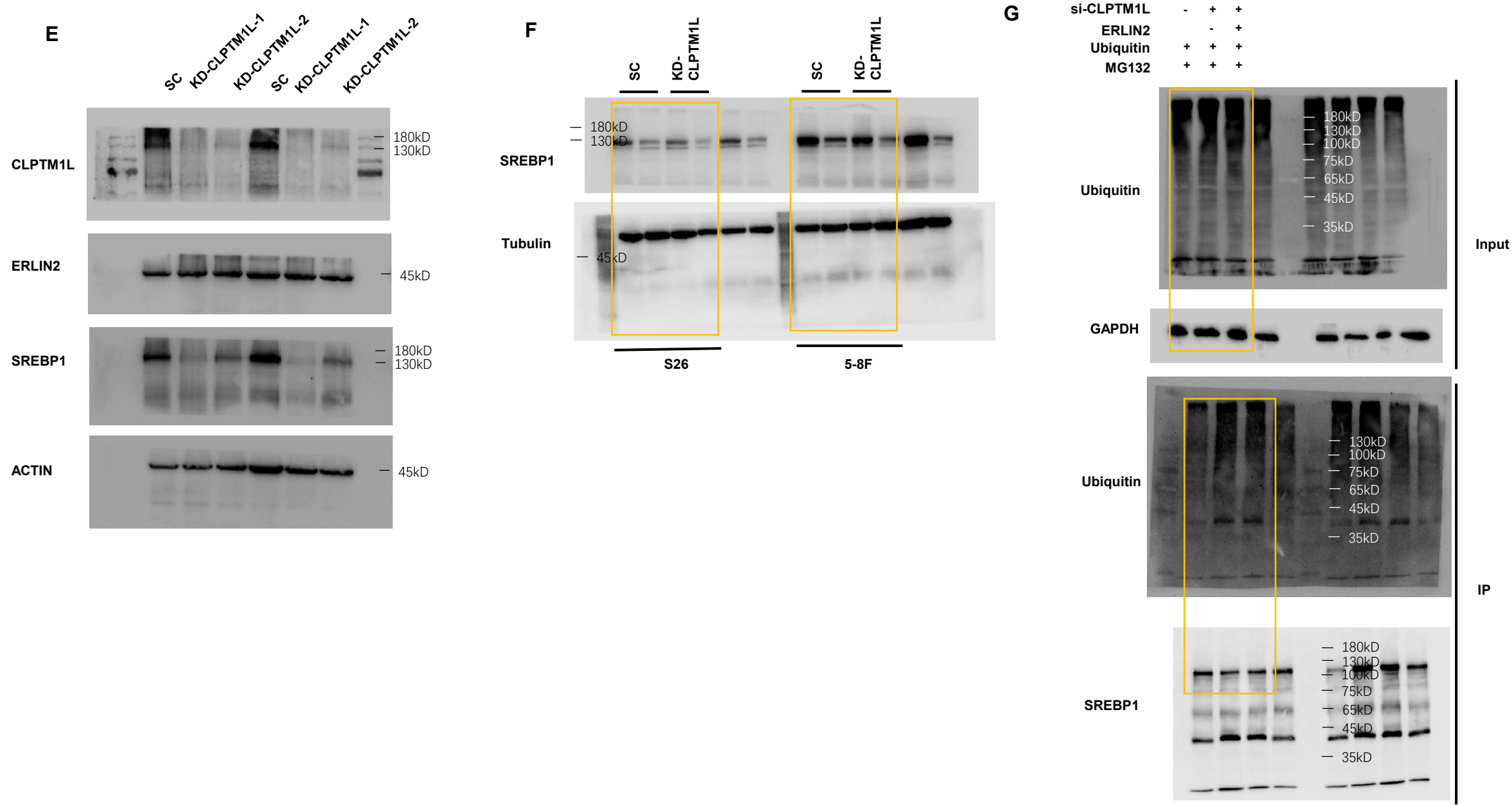

**A**

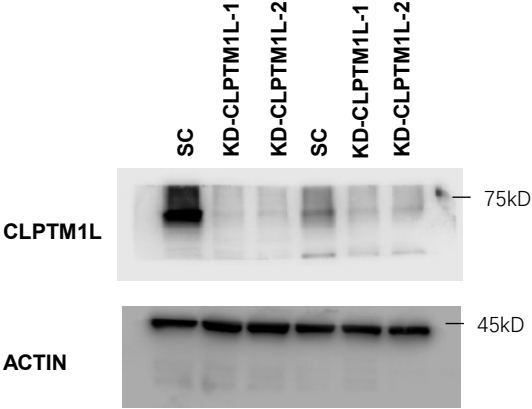

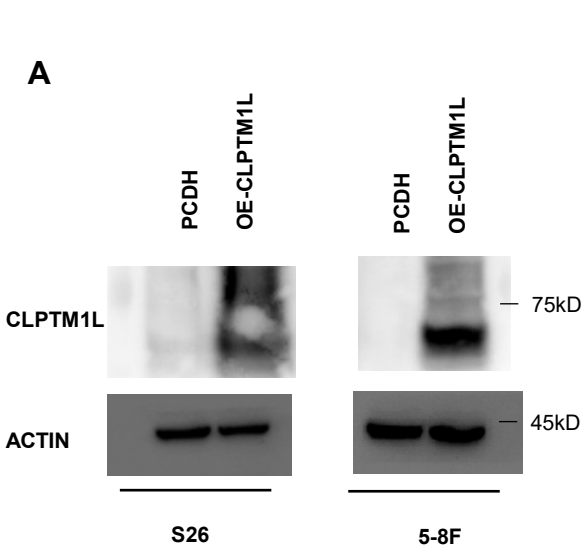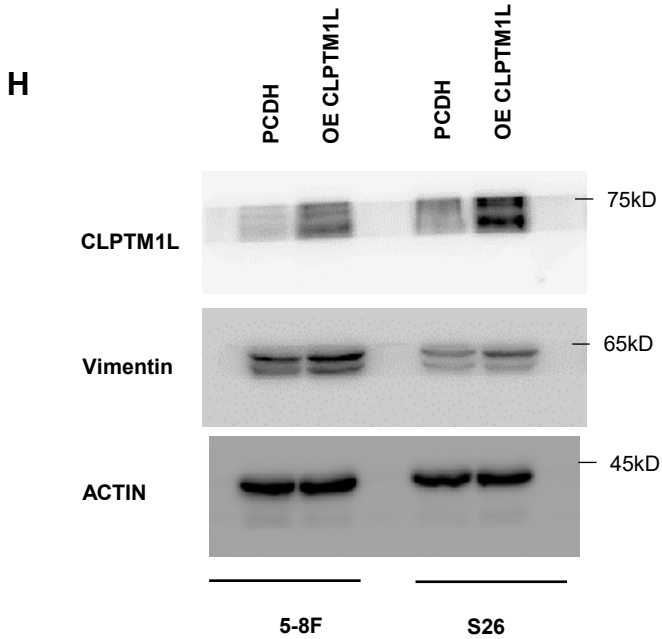

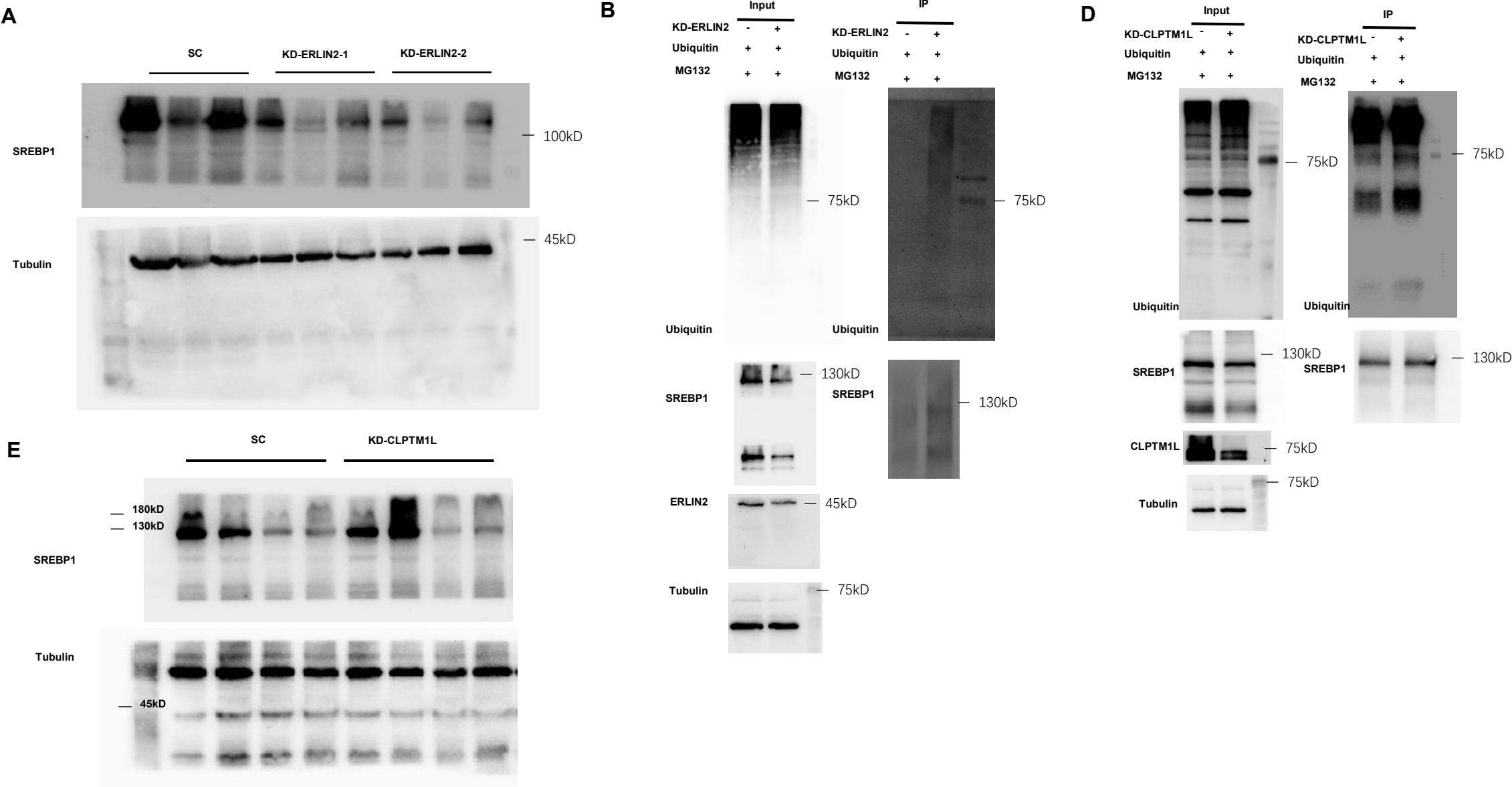

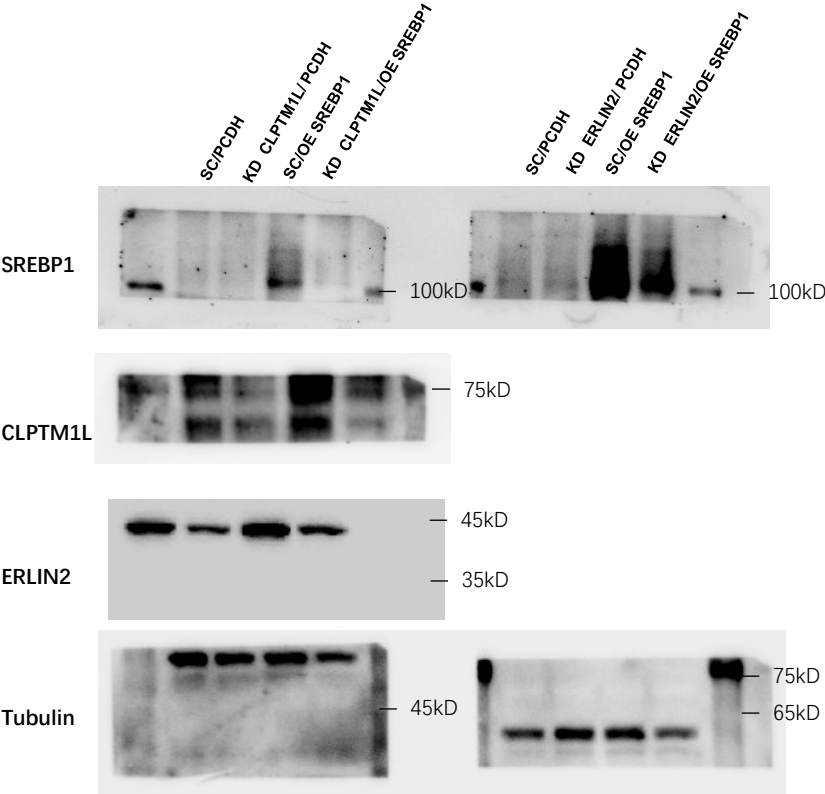

S26

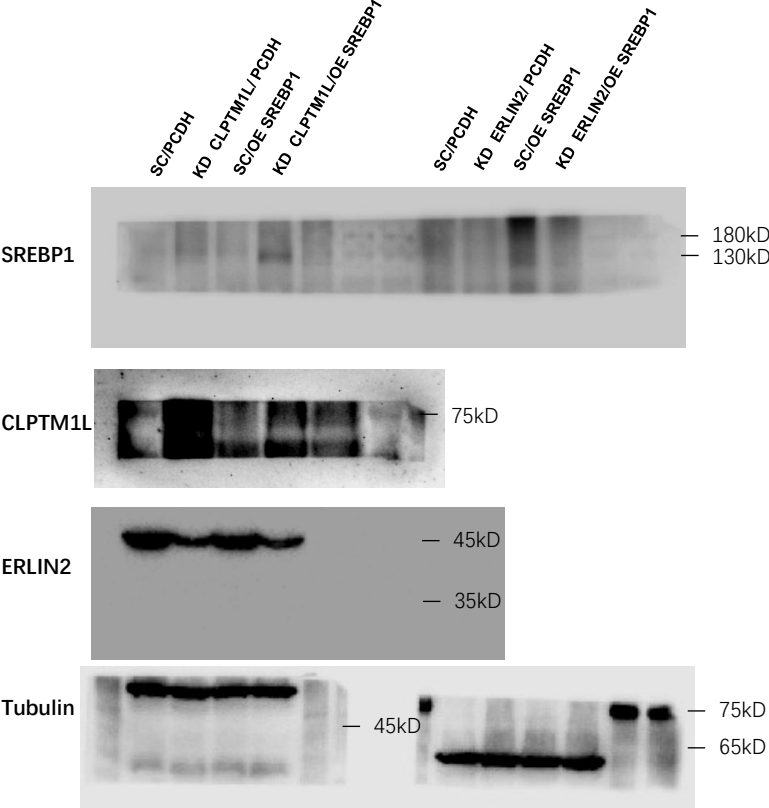

5-8F

Supplement: Supplementary file 3 — Original Western Data [file 41419_2025_7635_MOESM3_ESM.pdf]
